# Gut microbes and the liver circadian clock partition glucose and lipid metabolism

**DOI:** 10.1172/JCI162515

**Published:** 2023-09-15

**Authors:** Katya Frazier, Sumeed Manzoor, Katherine Carroll, Orlando DeLeon, Sawako Miyoshi, Jun Miyoshi, Marissa St. George, Alan Tan, Evan A. Chrisler, Mariko Izumo, Joseph S. Takahashi, Mrinalini C. Rao, Vanessa A. Leone, Eugene B. Chang

**Affiliations:** 1 Department of Medicine and; 2 Committee on Molecular Metabolism and Nutrition, The University of Chicago, Chicago, Illinois, USA.; 3 Department of General Medicine and; 4 Department of Gastroenterology and Hepatology, Kyorin University School of Medicine, Tokyo, Japan.; 5 Department of Animal & Dairy Sciences, University of Wisconsin–Madison, Madison, Wisconsin, USA.; 6 Department of Neuroscience and; 7 Howard Hughes Medical Institute, University of Texas Southwestern Medical Center, Dallas, Texas, USA.

**Keywords:** Metabolism, Fatty acid oxidation, Gluconeogenesis, Mouse models

## Abstract

Circadian rhythms govern glucose homeostasis, and their dysregulation leads to complex metabolic diseases. Gut microbes exhibit diurnal rhythms that influence host circadian networks and metabolic processes, yet underlying mechanisms remain elusive. Here, we showed hierarchical, bidirectional communication among the liver circadian clock, gut microbes, and glucose homeostasis in mice. To assess this relationship, we utilized mice with liver-specific deletion of the core circadian clock gene *Bmal1* via *Albumin*-*cre* maintained in either conventional or germ-free housing conditions. The liver clock, but not the forebrain clock, required gut microbes to drive glucose clearance and gluconeogenesis. Liver clock dysfunctionality expanded proportions and abundances of oscillating microbial features by 2-fold relative to that in controls. The liver clock was the primary driver of differential and rhythmic hepatic expression of glucose and fatty acid metabolic pathways. Absent the liver clock, gut microbes provided secondary cues that dampened these rhythms, resulting in reduced lipid fuel utilization relative to carbohydrates. All together, the liver clock transduced signals from gut microbes that were necessary for regulating glucose and lipid metabolism and meeting energy demands over 24 hours.

## Graphical abstract

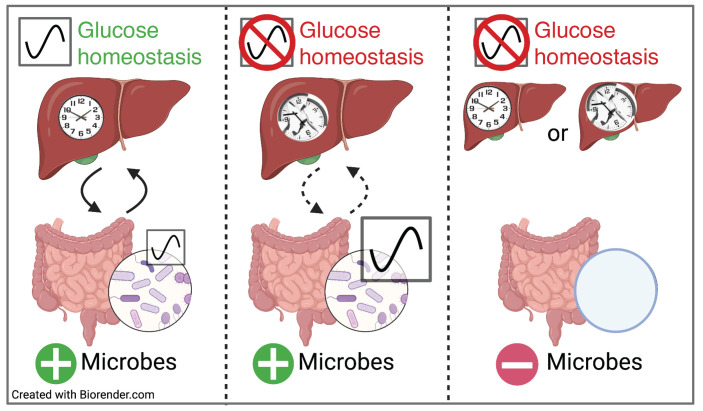

## Introduction

Circadian rhythms are essential to nearly all life, coordinating behavior and compartmentalizing physiological processes that govern energy balance over a 24-hour period (1). Self-sustaining and cell-autonomous, circadian rhythms serve to synchronize metabolism across active and inactive phases (2), including transitions between feeding and fasting, which is key for glucose regulation (3). Circadian disruption is linked to metabolic syndrome (4), and glucose dysregulation is a hallmark of the disease (5, 6). Gluconeogenesis (GNG), the endogenous production of glucose, is critical during periods of prolonged fasting (7). Along with the influence of endogenous hormones (8), both circadian rhythms and gut microbes drive GNG, and disruption of either leads to aberrant hepatic GNG (9, 10). Few mechanistic insights explain how these consequences arise; thus, understanding how these systems function in the context of metabolic homeostasis is of utmost importance.

The circadian clock molecular components are expressed in nearly all cells, forming an elegant feedback loop that organizes gene expression. *Bmal1* and *Clock* constitute the positive arm that activates gene transcription, including 2 *cryptochrome* genes (*Cry1–2*) and 3 *period* genes (*Per1–3*), which serve as the negative arm to repress *Bmal1* and *Clock*. This results in reduced expression of *Cry1–2* and *Per1–3*, allowing the positive arm to resume expression (11). Following genetic disruption of clock genes, metabolic networks become imbalanced, leading to disorders such as glucose intolerance and insulin resistance. While the central brain clock serves as the master regulator, peripheral tissue clocks exhibit unique oscillations and their disruption results in differential metabolic outcomes (reviewed in ref. 12). For instance, mice deficient in hepatic *Bmal1* uniquely display increased fasted glucose clearance, altered expression of hepatic GNG genes, and reduced functionality of mitochondria.

The gut microbiome is a key regulator of global host metabolism. Microbes vitally contribute to digestion and play a significant role in programming host energy balance (13–15). Their importance in metabolic homeostasis is established through the use of germ-free (GF) mice raised in complete absence of microbes (16). Environmental changes, such as diet, can rapidly affect microbial composition and functions that affect the host (13, 17). Disturbances in sleep also alter gut microbiome composition in both mouse and human models (18–20). Indeed, gut microbes are essential for normal energy balance and liver metabolic function, including hepatic GNG and glucose tolerance (9, 21, 22).

Gut microbes provide critical inputs that drive host circadian rhythms and metabolism, and exposure to high-fat diet disturbs these rhythms and functionality in mice (23, 24). High-fat diet also disrupts diurnal oscillations of microbial abundance and metabolite levels, while timed feeding can somewhat recover this effect (23, 25). Mice with global genetic mutations in circadian clock genes exhibit significantly altered microbial community profiles and loss of oscillations in specific taxa (18, 26, 27). Conversely, in absence of gut microbial cues, GF WT mice exhibit dampened rhythmicity in the expression of core circadian clock genes in both brain and liver, as well altered transcriptomic patterns in liver, small intestine, and white adipose tissue (23, 28). While the link among circadian rhythms, gut microbiota, and host metabolism is established, few mechanisms have been proposed to explain how these phenomena occur, how the circadian clock in specific metabolic organs such as the liver is involved, and how this affects global metabolic regulation. We hypothesized that hepatic GNG is driven by bidirectional interactions between the hepatic circadian clock and gut microbiota.

Here, using the liver-specific *Bmal1*-knockout mouse raised in both conventional and GF conditions, we demonstrated a hierarchy of signals between the gut microbiome and liver clock that coordinate hepatic GNG, fatty acid β-oxidation (FA β-Ox), and diurnal glucose homeostasis. We identified that regardless of microbial status, the liver clock is a primary driver of hepatic transcription, particularly of genes involved in the organization and function of key metabolic pathways. Additionally, the liver clock transduced timed signals from the gut microbiome to promote coordinated differential, oscillatory, and correlated transcription patterns in glucose and lipid metabolic pathways. When either of these components (host clock or microbes) were absent or dysfunctional, we observed a significant gain in oscillating hepatic transcripts that were disorganized. The absence of a functional clock also resulted in expansion of oscillations in specific fecal microbial abundances. Altogether, we revealed interactions between the liver circadian clock and gut microbes that aid in glucose and lipid homeostasis that governs whole-body metabolism and fuel utilization.

## Results

### Gut microbes are essential for liver circadian clock regulation of insulin-independent glucose clearance.

To assess the effects of a dysfunctional liver clock and gut microbes on mammalian host metabolism, we utilized male and female mice with liver-specific deletion of *Bmal1* gene expression generated by crossing *Bmal1^fl/fl^* mice with *Albumin-cre* mice (LKO mice), compared with control *Bmal1^fl/fl^* (WT) mice (29, 30). We confirmed *Bmal1* deletion in the liver, while expression in the brain remained intact (Supplemental Figure 1A; supplemental material available online with this article; https://doi.org/10.1172/JCI162515DS1). To assess the role of microbiota, we maintained both WT and LKO mice in conventional, specific-pathogen free (SPF) or GF conditions.

**Figure 1:**
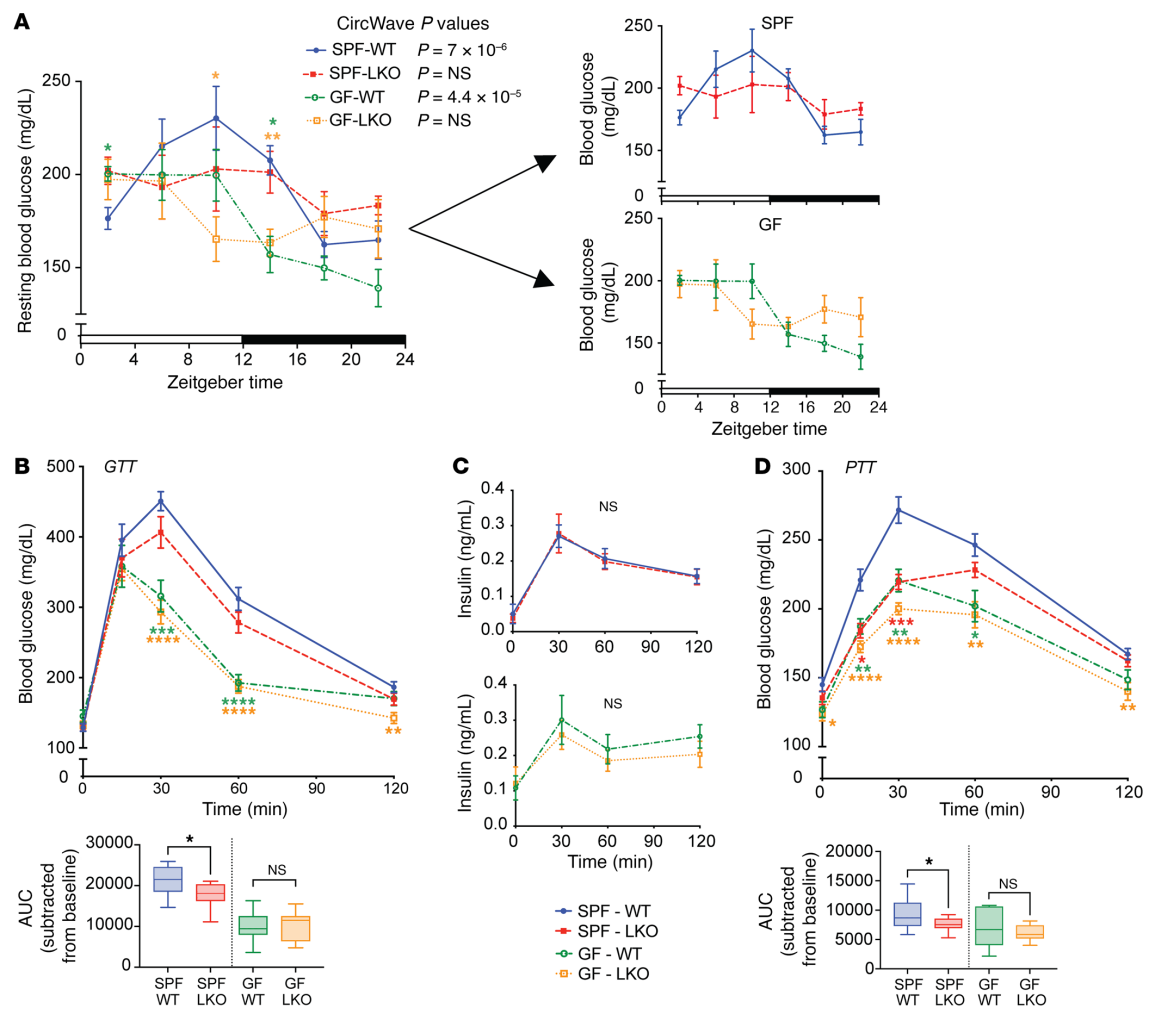
Gut microbes are essential for liver circadian clock–mediated glucose metabolism. (**A**) Resting blood glucose levels of SPF and GF WT and LKO male mice every 4 hours over 24 hours (*n* = 4-6/group/time point, SPF and GF groups also shown separately). CircWave statistics indicate significantly oscillating (*P* < 0.05) or not oscillating (*P* > 0.05) values. (**B**) GTT of SPF and GF WT and LKO male mice (*n* = 10–13/group). (**C**) Circulating insulin levels during GTT (*n* = 10–13/group). (**D**) PTT (*n* = 10–15/group) of SPF and GF WT and LKO male mice. Data are shown as the mean ± SEM. Lines in box plots represent the median, and whiskers represent the minimum and maximum, respectively. Two-tailed unpaired Welch’s *t* tests was performed between 2 groups; Brown-Forsythe and Welch’s ANOVA followed by Dunnett’s tests was performed between 3 or more groups. *P < 0.05, **P < 0.01, ***P < 0.001, ****P < 0.0001, relative to SPF WT. Graphs represent AUC normalized to baseline glucose.

SPF LKO male mice exhibited significantly increased body weight compared with that of SPF WT mice, while, in contrast, GF LKO mice exhibited a slight, but nonsignificant trend of decreased body weight relative to GF WT mice (Supplemental Figure 1B, left). This difference in body weight could not be explained by changes in gross liver or fat pad tissue weight (Supplemental Figure 1C). Weekly caloric consumption revealed similar patterns; SPF LKO mice ate slightly, but significantly, more than WT mice, while GF LKO mice ate slightly, but significantly less, than their WT counterparts (Supplemental Figure 1B, right). We then measured resting blood glucose levels every 4 hours over a 12:12 light/dark (LD) cycle and found that overall levels were not drastically different between groups in male mice (Figure 1A). However, analysis by CircWave revealed only WT mice, regardless of microbial status, exhibited significant oscillation of blood glucose levels, indicating that diurnal circulating blood glucose is driven by the liver clock and not gut microbes.

Given previous work by Lamia et al. (31), we performed an oral glucose tolerance test (GTT) and observed that male GF mice exhibited more rapid glucose clearance than their SPF counterparts (AUC, *P* < 0.05) (Figure 1B). We also observed a genotype-driven effect in SPF conditions where LKO mice cleared glucose significantly faster than WT mice. Circulating insulin levels during the GTT were not significantly different between WT and LKO mice in either SPF or GF conditions, suggesting that insulin secretion was not affected by liver clock functionality (Figure 1C). We next interrogated insulin sensitivity by intraperitoneal insulin tolerance test (ITT) on SPF and GF WT and LKO male mice and found no difference in insulin sensitivity between genotypes in either microbial condition (Supplemental Figure 1D), suggesting that differences in male *Bmal1*-dependent glucose clearance are insulin independent.

These data suggest that the liver circadian clock imparts an insulin-independent effect on glucose clearance that is dependent on the presence of gut microbes.

### Circadian clock– and microbiome-driven GNG is liver specific and requires in vivo signals.

To interrogate the role of the liver clock on GNG, we performed an intraperitoneal pyruvate tolerance test (PTT). We found that GF mice exhibited significantly lower GNG rates than their SPF counterparts, regardless of genotype (Figure 1D). We also observed that LKO mice exhibited reduced GNG in SPF conditions, while no differences between genotypes in GF conditions were evident, indicating that the liver clock mediates GNG via gut microbes.

To determine whether reduced GNG is liver clock–specific, we performed PTT on mice lacking a functional core brain clock by *CamkIIa*-*cre* forebrain-specific *Bmal1* knockout, while peripheral clocks remain intact (32, 33) (Supplemental Figure 1A). We found no difference in GNG rates between WT and forebrain-*Bmal1*-KO mice (Supplemental Figure 1E), indicating that clock-mediated changes in GNG are specific to hepatic *Bmal1* and the liver clock. This result corroborates previous evidence that the liver clock, specifically hepatic *Bmal1*, is somewhat autonomous from the core clock located in the brain (34).

Because both GNG and glycogenolysis are costimulated during periods of prolonged fasting, we measured glycogen levels in liver samples collected from each group every 4 hours over 24 hours. We observed no difference between SPF groups at any time point, and glycogen content was only elevated in GF WT mice relative to LKO mice at zeitgeber time 22 (ZT22; 4 am) (Supplemental Figure 1F). In all groups, liver glycogen levels exhibited significant and remarkably similar oscillations over a 24-hour period, as evident by CircWave statistics. These data indicate that hepatic glycogenolysis is not a major contributor to either the microbe- or clock-mediated effect on the observed glucose homeostasis phenotype, further supporting that GNG is the major contributor.

Finally, we examined glucose production in primary hepatocytes isolated from SPF and GF WT and LKO mice ex vivo following stimulation with GNG-inducing substrates in glucose-free media. We found no difference in glucose production in media between groups following GNG stimulation with a cell permeable cAMP analog (cPT-cAMP) (Supplemental Figure 1G). This suggests that the cellular machinery to perform GNG is not affected by liver clock functionality or prior exposure to gut microbes and that additional in vivo signals are necessary for liver clock– and microbe-mediated GNG.

In addition to the above experiments performed using male mice, we also performed several of the same experiments using female mice to investigate the potential role of sex. Interestingly, we did not observe any differences between genotype in female mice for body weight or food intake (Supplemental Figure 2A) and little difference in diurnal blood glucose (Supplemental Figure 2B). Additionally, GTT in female mice revealed no significant differences in AUC (Supplemental Figure 2C), suggesting that liver clock–mediated glucose clearance is in part driven by sex. We also performed PTT of female SPF WT and LKO mice and observed no difference in GNG (Supplemental Figure 2D). Given these results indicating that liver clock–mediated glucose clearance and GNG are male specific, we proceeded with only male mice for the duration of the study.

Overall, we found a microbiota-dependent effect of liver circadian clock–mediated GNG in male mice that requires real time in vivo signals.

### Gut microbes are necessary, but not sufficient, for liver clock–mediated GNG.

**Figure 2:**
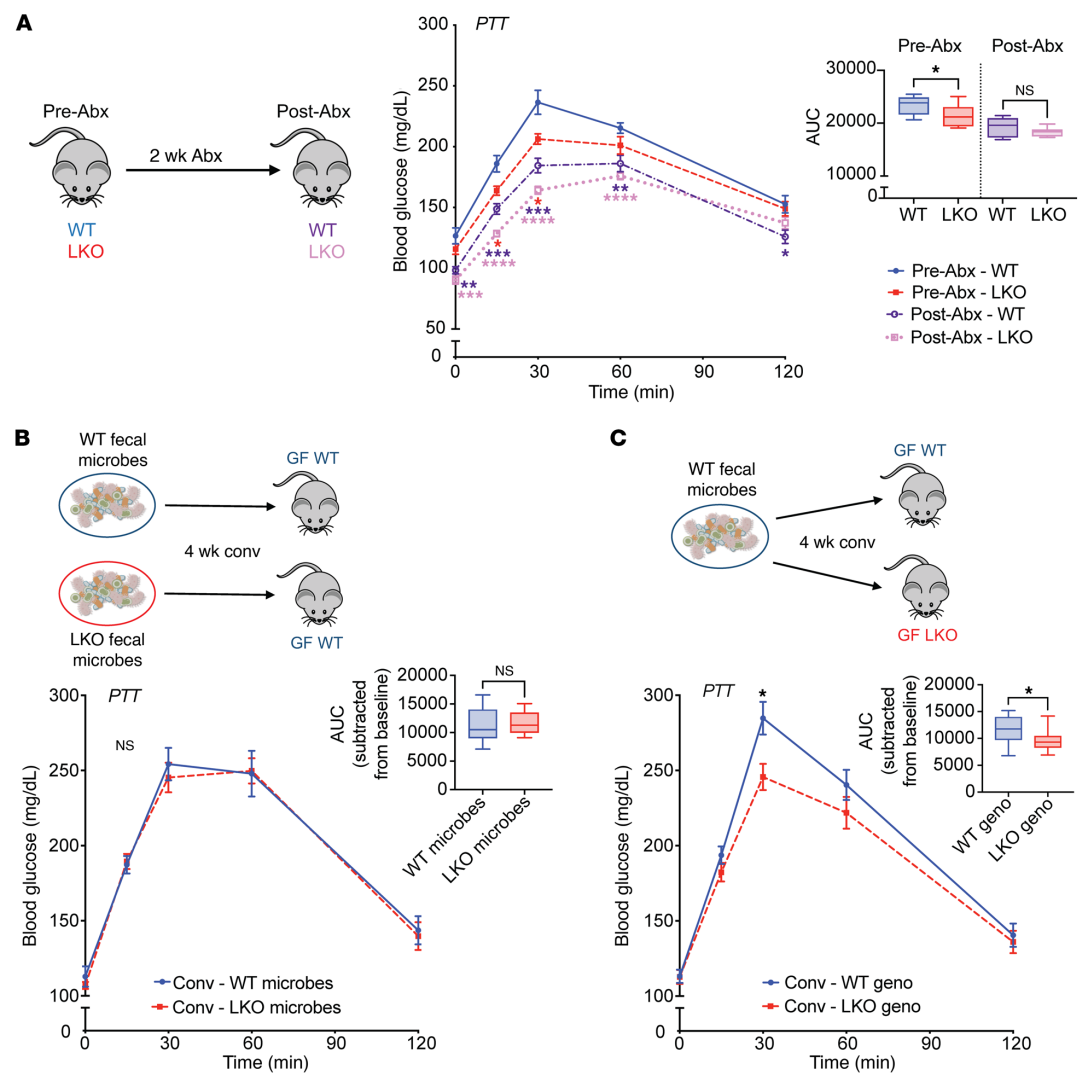
Modulation of gut microbes can both eliminate and restore liver clock–mediated GNG. (**A**) PTT in WT and LKO male mice before (Pre-Abx) and after (Post-Abx) daily antibiotic treatment for 2 weeks (*n* = 12–13/group). The graph represents the AUC. (**B**) PTT in GF WT male mice conventionalized with fecal microbes from SPF WT or LKO male mice (*n* = 11–12/group). (**C**) PTT in GF WT and LKO male mice conventionalized with fecal microbes from SPF WT male mice (*n* = 15–16/group). Inset graphs represents AUC normalized to baseline glucose. Data points represent mean ± SEM. Lines in box plots represent the median, and whiskers represent the minimum and maximum, respectively. Two-tailed unpaired Welch’s *t* tests were performed between 2 groups; Brown-Forsythe and Welch’s ANOVA followed by Dunnett’s tests were performed between 3 or more groups. *P < 0.05, **P < 0.01, ***P < 0.001, ****P < 0.0001, relative to Pre-Abx WT.

To investigate the role of gut microbes, we performed PTT in adult SPF WT and LKO mice both before (Pre-Abx) and after (Post-Abx) acute elimination of gut microbes via daily antibiotic treatment for 2 weeks. We also confirmed significant bacterial reduction, as determined by 16S gene copy number in Post-Abx stool via RT-PCR (Supplemental Figure 3A). While PTT of Pre-Abx mice mimicked our SPF results in Figure 1D, we observed no differences in GNG between genotypes Post-Abx, as measured by AUC, closely resembling outcomes in GF mice (Figure 2A). This supports the hypothesis that gut microbial cues are essential for in vivo coordination of GNG mediated by the liver clock.

Next, we conventionalized adult GF C57BL/6J WT mice via fecal microbiota transplant from either WT or LKO SPF donor mice and performed PTT (Figure 2B). Here, we did not observe significant differences between recipient groups. This suggests that gut microbes selected by the presence or absence of a functional liver clock alone were insufficient to transfer the GNG phenotype, implying that genotype is the primary driver of GNG. We then performed a different microbiota transplant in which we conventionalized GF WT or LKO recipient mice with identical WT donor fecal microbes (Figure 2C). We observed significantly reduced GNG in LKO mice relative to that in WT recipients, indicating that restoration of the LKO SPF phenotype requires both gut microbes and genetic absence of a liver clock.

Given that gut microbes directly modulate GNG via the liver clock, we sought to determine whether loss of *Bmal1* affects microbiota community membership. Cohousing animals can normalize microbiota differences and mask the effect of genotype, largely due to the coprophagic tendencies of mice; therefore, we compared SPF WT and LKO mice in a mixed housing scenario (WT + LKO) with mice separated by genotype at weaning (WT + WT vs. LKO + LKO) (Supplemental Figure 3B) until 12 weeks of age. Regardless of housing, we detected no significant differences in relative abundance of 16S rRNA gene amplicon sequence variants (ASVs) belonging to either dominant phyla or less abundant phyla (Supplemental Figure 3, C and D) or in overall community membership via metrics of β-diversity (Supplemental Figure 3E).

Taken together, liver clock functionality does not impact overall gut microbiota community assemblage; however, the liver clock serves as the primary driver of GNG, while microbes are key secondary drivers. This suggests a concurrent interaction between gut microbes and the liver clock that requires real-time microbial signals to mediate GNG.

### The liver clock drives oscillations of specific microbiota community members.

**Figure 3:**
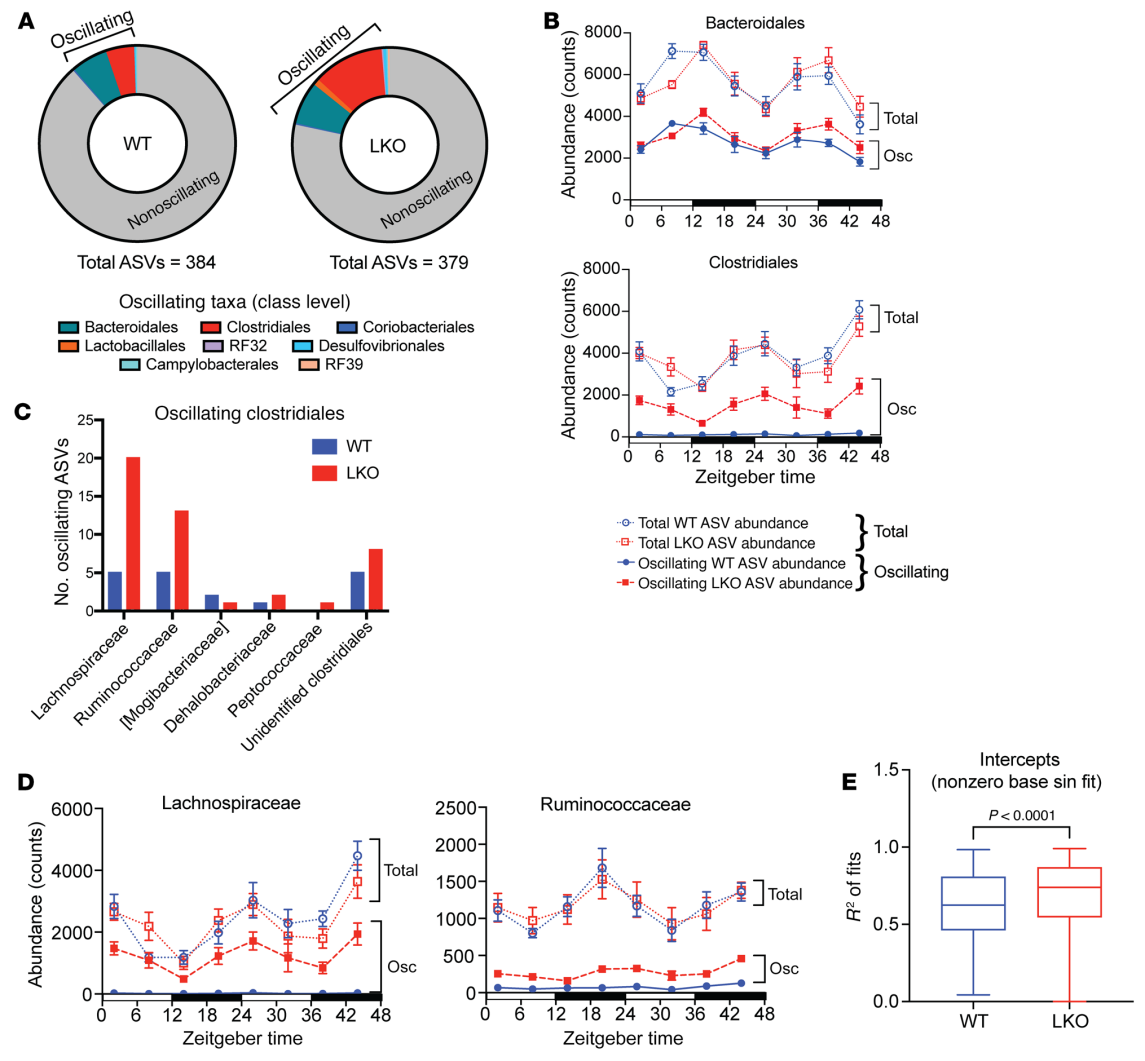
Liver circadian clock drives unique patterns of oscillations in microbial abundance. 16S rRNA gene sequencing of stool from SPF WT and LKO male mice every 6 hours over 48 hours via repeat collection (*n* = 7–8/group). (**A**) Proportion of nonoscillating (gray area) versus significantly oscillating (colored areas) amplicon sequence variants (ASVs) identified via eJTK (GammaBH < 0.05). Oscillating (Osc) ASVs were divided by taxonomic class. (**B**) Abundance counts of total versus oscillating ASVs within Bacteroidales and Clostridiales classes. (**C**) Number of oscillating Clostridiales ASVs at the family level in WT and LKO mice. (**D**) Abundance counts of total versus oscillating ASVs within Lachnospiraceae and Ruminococcaceae families. (**E**) *R*^2^ values of nonzero base sinusoidal fits of log ratios at each time point relative to ZT2. Data represent mean ± SEM. Lines in box plots represent the median, and whiskers represent the minimum and maximum, respectively. Two-tailed paired *t* test was performed.

Global deletion of *Bmal1* in mice has been previously shown to alter diurnal oscillations of fecal microbiota (26). Thus, we inquired whether liver clock functionality affected diurnal oscillations of microbiota in feces collected from WT and LKO male and female mice every 6 hours over 2 consecutive 24-hour light/dark periods (Supplemental Figure 4A). We found that absolute 16S gene copy number was similar between WT and LKO mice across all time points for both sexes (Supplemental Figure 4B and Supplemental Figure 5A). We next utilized empirical Jonckheere-Terpstra-Kendall CYCLE (eJTK) to identify significantly oscillating ASVs. Here, we discovered that male LKO mice exhibited nearly twice the number of oscillating ASVs compared with WT mice (Figure 3A and Supplemental Figure 4C). The influx of unique oscillating microbes in LKO mice was mostly attributed to ASVs identified to class Clostridiales. Conversely, we observed little difference in the number of oscillating Bacteroidales ASVs, suggesting that LKO mice may specifically impact diurnal oscillations of Clostridiales taxa. Interestingly, we observed little difference in the number of oscillating ASVs between female WT and LKO mice (Supplemental Figure 5, B and C).

Aside from proportion, the relative abundance of oscillating ASVs was also substantially greater in LKO relative to WT mice (Supplemental Figure 4D). We identified that while overall abundance of total versus oscillating Bacteroidales and Clostridiales ASVs was not different between WT and LKO mice, only Clostridiales exhibited increased abundance of oscillators in LKO mice (Figure 3B). We then examined oscillating Clostridiales at the family level and identified that Lachnospiraceae and Ruminococcaceae accounted for the gain of uniquely oscillating ASVs in LKO mice (Figure 3C). While the total abundance of either family was not different between genotypes, the abundance of individual oscillating ASVs greatly increased within LKO mice (Figure 3D). Interestingly, among the ASVs annotated to species, *Lactobacillus murinus* similarly appeared to gain oscillation in LKO mice, while total abundance remained constant (Supplemental Figure 4E).

In addition to the eJTK oscillation analysis, we also examined log ratios, which are not sensitive to microbial load and rely on more reproducible reference frames (35, 36). We generated and ranked log ratios for all ASVs at each time point relative to ZT2 (Supplemental Figure 6A). LOESS-fitting plots of the log ratios showed more robust oscillation patterns in LKO mice when grouped at the family level as compared with WT mice (Supplemental Figure 6B), particularly for Ruminococcaceae, Lachnospiraceae, and Lactobacillaceae, confirming our eJTK results. Other taxonomic groups also exhibited a more robust oscillation pattern in LKO mice compared with WT mice, including Muribaculaceae and Erysipelotrichaceae. Finally, application of a nonzero sinusoidal fit analysis identified that log ratios of ASVs in LKO mice exhibit a significantly greater sinusoidal fit than WT mice (*P* < 0.0001), further confirming our previous eJTK oscillation analysis of relative abundance data (Figure 3E).

Altogether, despite a lack of changes in overall microbial profiles, loss of a functional liver clock drives a signature of increased diurnal oscillations in specific stool microbiota community members, particularly those belonging to the class Clostridiales.

### The liver clock is the main driver of hepatic transcriptomes, secondarily influenced by gut microbes.

**Figure 4:**
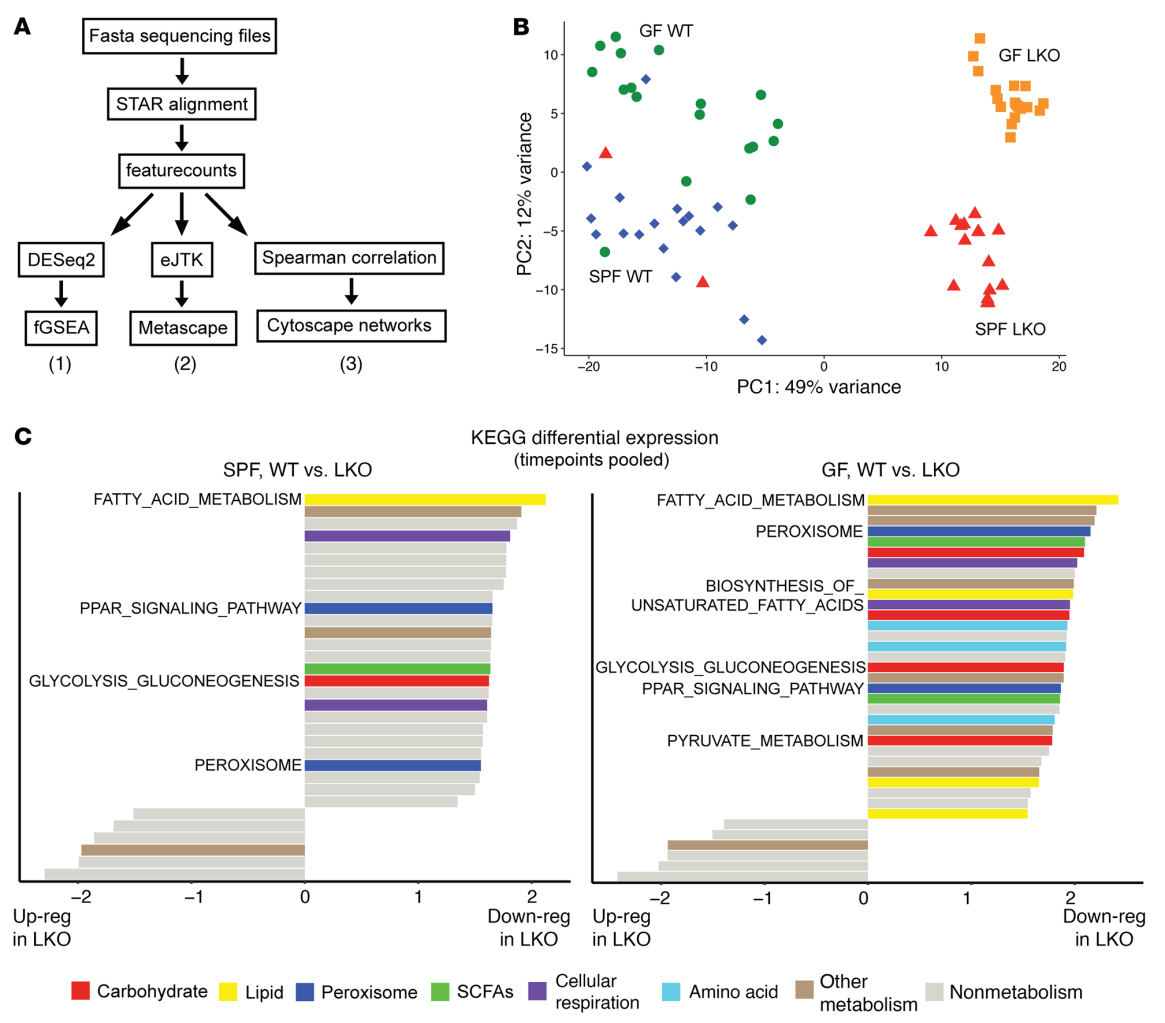
Metabolic pathway gene expression is downregulated in absence of a liver clock across time, regardless of microbial status. Transcriptome analysis of liver samples collected every 4 hours over 24 hours (ZT2, -6, -10, -14, -18, and -22) from SPF and GF WT and LKO male mice (*n* = 3/time point/group). (**A**) Data analysis workflow, demonstrating 3 arms of analysis: 1, differential expression; 2, oscillation; and 3, network coexpression. (**B**) Principal component analysis of transcriptome profiles; all samples within each group were pooled. (**C**) Differentially regulated KEGG pathways between WT and LKO mice within SPF and GF groups; all samples within each group were pooled. Metabolic pathways are colored (see key),and nonmetabolic pathways are colored gray. Bars to the right of the midline plot represent pathways downregulated in LKO mice compared with WT mice; bars to the left represent pathways upregulated in LKO mice compared with WT mice.

Given that liver clock and gut microbes regulate GNG, we sought to determine whether this and other molecular metabolic pathways were effected. We performed RNA-Seq of liver samples collected every 4 hours over a 12:12 light/dark cycle from SPF and GF WT and LKO male mice. These data were analyzed using 3 approaches (Figure 4A). First, principle component analysis of pooled samples revealed distinct separation by genotype along PC1 and microbial status along PC2 (49% and 12% of variance, respectively) (Figure 4B). Similar patterns were observed when samples were divided by time point, demonstrating consistency across the 24-hour period (Supplemental Figure 7A). This suggests a hierarchy in which liver clock functionality (genotype) is the primary driver of the hepatic transcriptome, while the gut microbiome serves as a secondary driver.

We next performed differential gene expression analysis via DESeq2 and fast gene set enrichment analysis (fGSEA) to identify molecular pathways affected by the liver clock and/or gut microbes (Figure 4A, approach 1). Analysis using Kyoto Encyclopedia of Genes and Genomes (KEGG; https://www.genome.jp/kegg/) revealed that nearly all differentially expressed metabolic pathways were downregulated in LKO compared with WT mice, regardless of microbial status (Figure 4C). However, GF conditions exhibited more significantly downregulated metabolic pathways in LKO than SPF conditions. This may suggest that presence of gut microbes results in less permissive compensatory metabolic pathway transcriptional regulation in absence of *Bmal1*; that is, certain *Bmal1*-driven effects only emerge in absence of microbes. Differential analysis within collection time points revealed nearly identical patterns of overall downregulation of metabolic pathways to the fGSEA analysis (Supplemental Figure 7B). Importantly, “Glycolysis_Gluconeogenesis” was downregulated in SPF LKO mice compared with WT mice (Figure 4C, left), confirming our evidence that GNG was impaired in SPF LKO mice (Figure 1D). We also observed downregulation of GNG pathways, including “Pyruvate_Metabolism,” in GF LKO mice compared with WT mice (Figure 4C, right), while our physiological evidence revealed no difference in overall GNG output between GF WT and LKO mice (Figure 1D). This suggests that while differences in transcription between genotypes are similar in GF and SPF conditions, lack of additional signals (presumably microbial) contribute to reduced GNG in GF conditions regardless of genotype.

Apart from GNG, we observed LKO resulted in downregulation of several metabolic pathways involved in FA and lipid metabolism, including “Fatty_Acid_Metabolism,” “PPAR_Signaling_Pathway,” “Peroxisome,” and “Biosynthesis_of_Unsaturated_Fatty_Acids” (Figure 4C). This corroborates previous findings that lipid metabolic gene expression, as well as triglyceride, cholesterol, and steroid metabolism, is downregulated in liver clock–deficient mice compared with WT mice (34, 37–39). The regulation of GNG and FA metabolism are intricately tied; under fasting conditions, FA β-Ox is activated to provide acetyl-CoA to generate ATP, which sustains the conversion of pyruvate and other GNG substrates into glucose (40). Thus, the regulation of these two processes is closely linked and changes in one directly affect the other (41).

**Figure 5:**
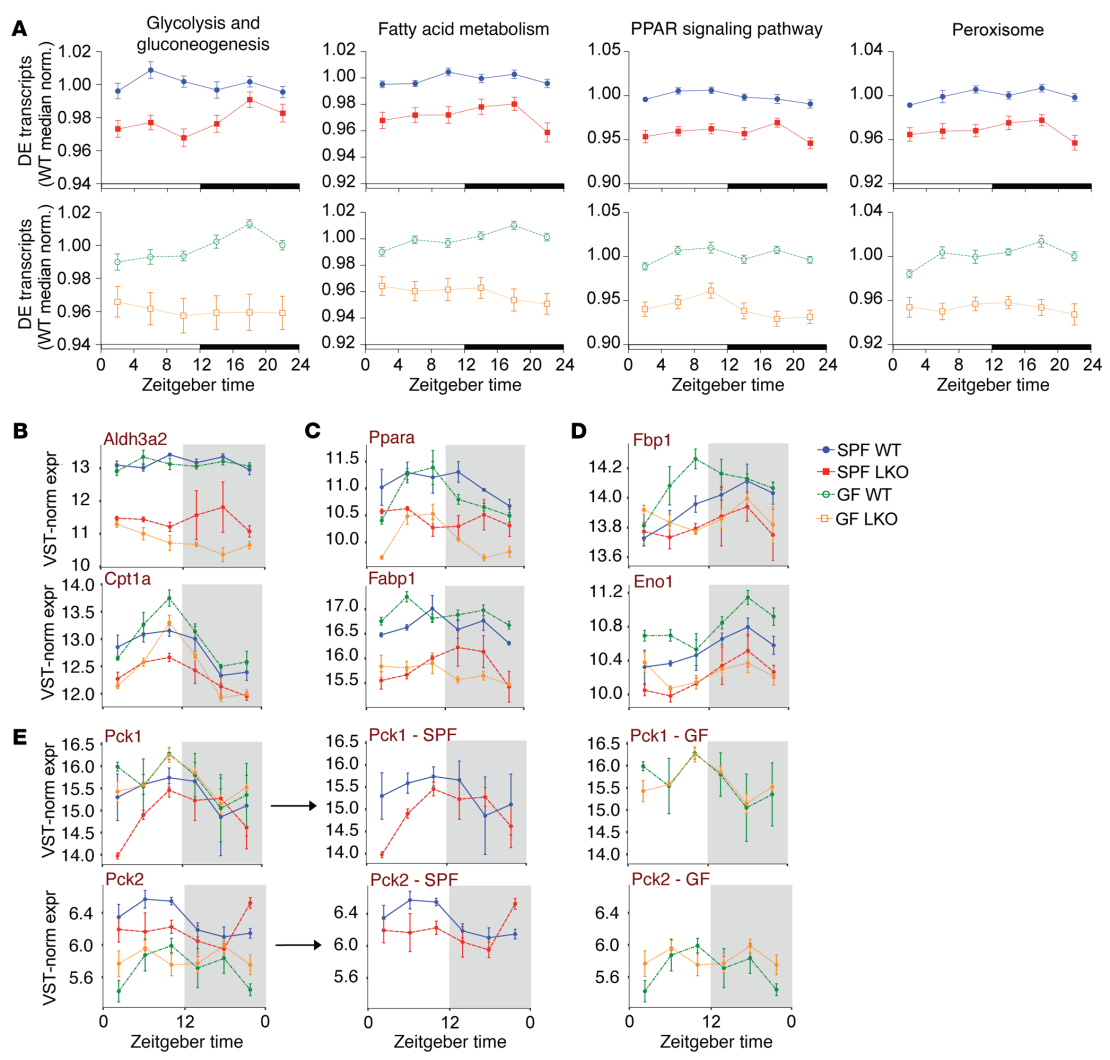
Gut microbes work through the liver clock to impart unique expression patterns of key gluconeogenic genes. Diurnal transcriptome analysis of liver samples collected every 4 hours over 24 hours from SPF and GF WT and LKO male mice (*n* = 3/time point/group) maintained in 12:12 LD (ZT2, -6, -10, -14, -18, and -22). (**A**) WT-median-normalized expression of differentially expressed (DE) genes within identified KEGG pathways. (**B**–**E**) VST-normalized expression of leading-edge genes in FA metabolism (**B**), PPAR signaling (**C**), and GNG (**D** and **E**) D between SPF and GF WT and LKO mice, with the exception of *Pck1* and *Pck2* (**E**), which are only differentially expressed in the SPF group (SPF and GF groups shown separately). Data represent mean ± SEM.

Given this differential regulation, we plotted median-normalized expression for all leading-edge transcripts within relevant pathways, confirming marked downregulation in LKO mice compared with WT mice in both SPF and GF conditions (Figure 5A and Supplemental Figure 7C). We next examined expression of specific transcripts within these pathways, in particular key transcripts involved in FA metabolism (Figure 5B; aldehyde dehydrogenase 3 family member A2 [*Aldh3a2*]; carnitine palmitoyltransferase 1A [*Cpt1a*]), PPAR signaling (Figure 5C; *Ppara;* fatty acid binding protein 1 [*Fabp1*]), and GNG (Figure 5, D and E; fructose-bisphosphate 1 [*Fbp1*]; enolase 1 [*Eno1*]; phosphoenolpyruvate carboxylase 1/2 [*Pck1/2*]). Expression was greatly reduced in many of these transcripts in LKO mice compared with that in WT mice regardless of microbiota status (Figure 5, B–D). However, expression of the key rate-limiting GNG enzymes *Pck1* and *Pck2* exhibited more nuanced patterns (Figure 5E). *Pck1* revealed reduced expression in SPF LKO mice compared with WT mice, while both GF groups exhibited expression levels similar to SPF WT mice. Conversely, *Pck2* was considerably reduced in SPF LKO mice compared with WT mice, and both GF groups were reduced compared with SPF, mirroring our PTT results (Figure 1D). Thus, *Pck2* may be key for liver clock and microbially mediated effects on GNG, while *Pck1* is important only under SPF conditions.

In summary, the liver clock is the primary driver of transcriptome-wide differential regulation, particularly for key metabolic pathways, such as GNG, which are downregulated in absence of a liver clock. However, gut microbes provide additional cues to modulate expression, including LKO-driven expression of the rate-limiting GNG enzyme *Pck2*.

### Liver clock and gut microbes drive unique hepatic transcriptome oscillations.

**Figure 6:**
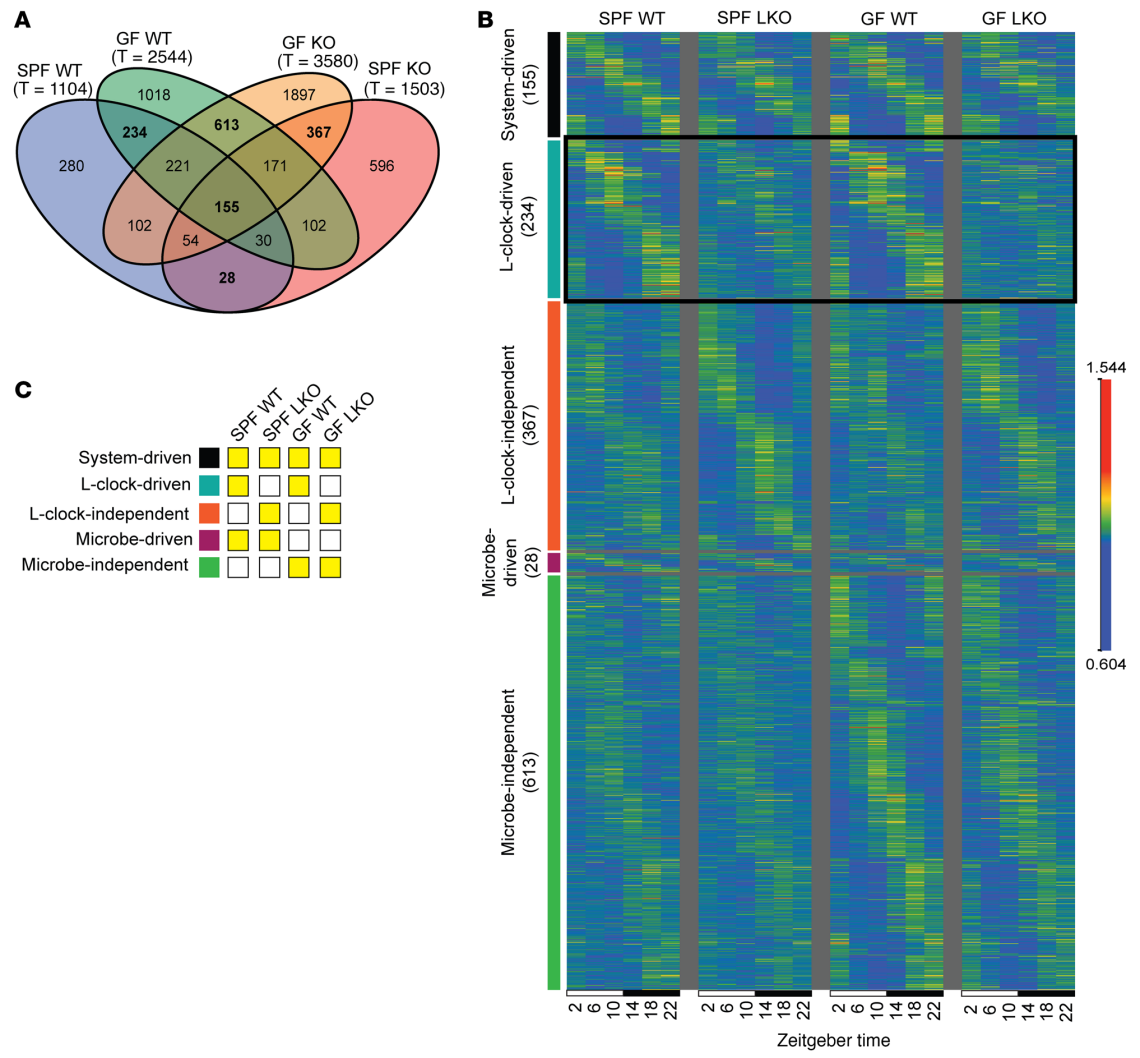
Liver clock and gut microbes drive unique hepatic transcriptome oscillations. Diurnal transcriptome analysis of liver samples collected every 4 hours over 24 hours from animals maintained in 12:12 LD (ZT2, -6, -10, -14, -18, -22) from SPF and GF WT and LKO male mice (*n* = 3/time point/group). (**A**) Venn diagram of significantly oscillating transcripts across each group; total number of oscillating transcripts are under each group title. Oscillating transcripts were identified via eJTK (GammaBH < 0.05). Bold numbers are visualized in Figure 5B. (**B** and **C**) Expression of significantly oscillating transcripts that are system driven, liver clock driven or independent, and microbe driven or independent (**B**). Expression was normalized by median, and transcripts were ordered by time of maximum expression and phase; the key indicates which transcripts are depicted with yellow (**C**).

Given previous findings (42), we identified all significantly oscillating transcripts within each group via eJTK (Figure 4A, approach 2). SPF LKO mice exhibited more oscillating transcripts than WT mice (1,503 vs. 1,104), and GF LKO mice exhibited a similar increase relative to GF WT mice (3,580 vs. 2,544) (Figure 6A). Only 155 transcripts were oscillating in all groups, while many were uniquely oscillating in a single group. This emergence of unique oscillators in the absence of a functional liver clock mirrored the pattern observed in ASV relative abundance in repeat-collected stool (Figure 3A). These data reveal that loss of key drivers (i.e., the liver clock or gut microbes) results in emergence of unique oscillatory elements both in the hepatic transcriptome and gut microbiota community, which may contribute to a loss in metabolic homeostasis in vivo.

We then compared oscillating gene expression patterns. While SPF WT transcripts exhibited clear diurnal patterns, oscillations were significantly dampened in LKO transcripts, regardless of microbial status (Supplemental Figure 8A). Conversely, the oscillation patterns were well-preserved in GF WT mice, reinforcing that the liver clock is the main driver of core oscillating transcripts. We next partitioned oscillating transcripts into categories reflecting those that are system driven (i.e., exhibit a significant oscillation regardless of liver clock or microbial status), liver clock driven, liver clock independent, microbe driven, and microbe independent (Figure 6, B and C). System-driven transcripts exhibited a conserved oscillation pattern across groups, while transcripts in the other categories exhibited dampened oscillations. For example, microbe-independent transcripts demonstrated robust oscillation across GF groups, with clear dampening of oscillation across SPF groups. Interestingly, the only subset of transcripts that exhibited a unique pattern was the liver clock–driven oscillating transcripts (Figure 6B, bolded box). Whereas both WT groups exhibited robust oscillations in liver clock–driven transcripts, LKO groups exhibited severe dampening. Between LKO groups, the SPF LKO group displayed a more preserved organization of oscillating transcripts, while the highest level of disorganization was observed in the GF LKO group relative to the WT group. This suggests that the liver clock and gut microbes impart combinatorial action on the temporal organization of specific liver clock–driven diurnal hepatic gene expression.

Overall, the liver clock and gut microbes independently impart a unique effect on the oscillating transcriptome, and absence of both drivers results in further disorganization of oscillating transcripts.

### Liver clock and gut microbes exhibit individual and combinatorial influences on hepatic GNG and FA metabolic pathway expression.

**Figure 7:**
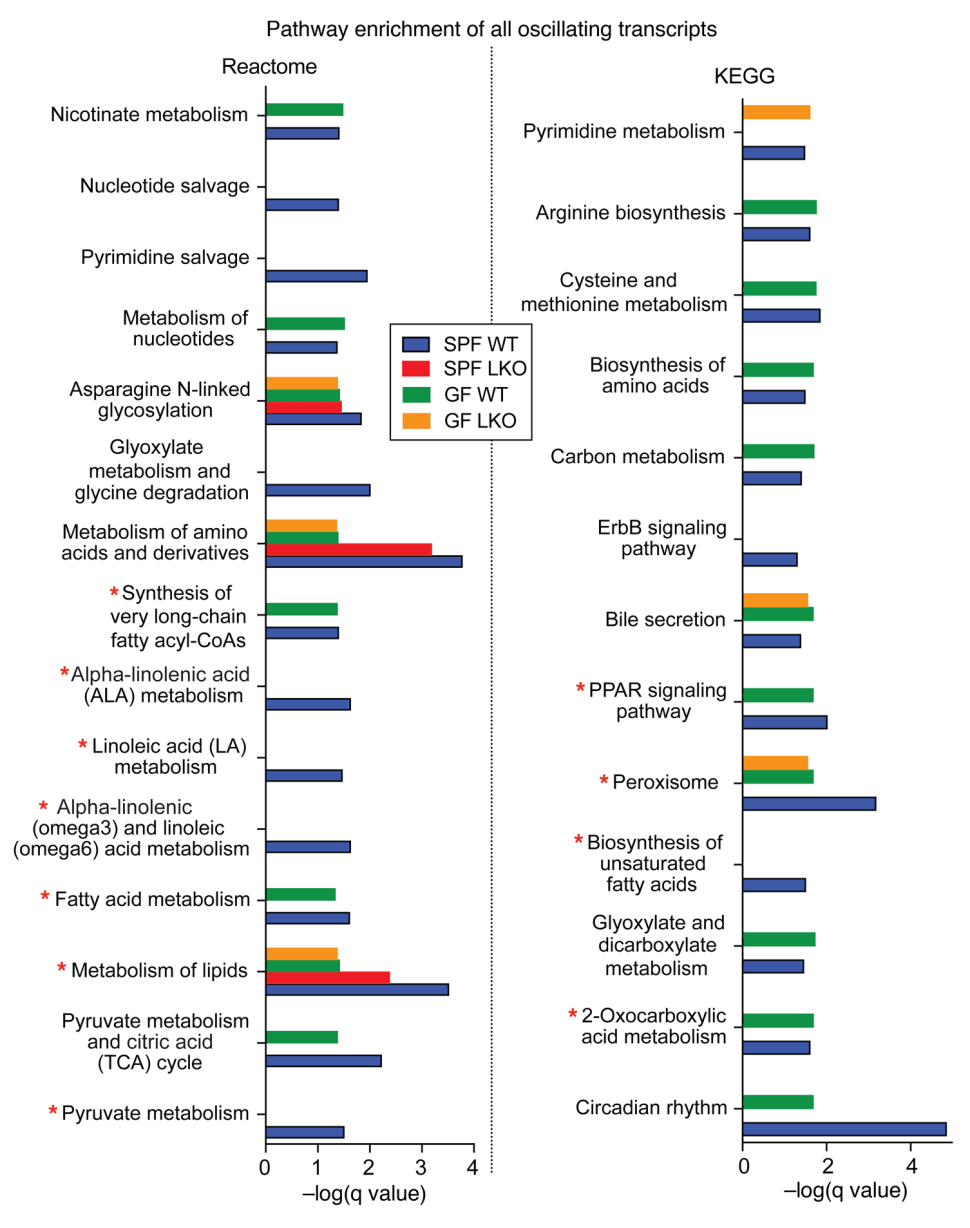
Liver clock and gut microbes uniquely impact functional pathway enrichment of oscillating hepatic transcripts. Diurnal transcriptome analysis of liver samples collected every 4 hours over 24 hours from SPF and GF WT and LKO male mice (*n* = 3/time point/group) maintained in 12:12 LD (ZT2, -6, -10, -14, -18, and -22). Reactome and KEGG pathways significantly enriched by oscillating transcripts within each group. A subset of pathways enriched in SPF WT oscillating genes (*q* < 0.05). A lack of bar indicates lack of significance for that group/pathway (*q* > 0.05). Pathways marked with red star are addressed in the text.

Next, we applied Metascape (43) to statistically determine which pathways were significantly enriched in each group of oscillating transcripts (*q* < 0.05, summarized in Supplemental Table 1). In examining pathways enriched in SPF WT oscillating liver transcripts relative to other groups, we observed that each factor (liver clock and gut microbiota) elicited similar levels, reduced levels, or total absence of enrichment (Figure 7). Both SPF and GF WT groups exhibited similar enrichment across many pathways, supporting our finding that oscillating hepatic transcriptome patterns are primarily driven by the liver clock. However, we observed enrichment of Reactome “Pyruvate Metabolism” only in SPF WT oscillating transcripts. This loss of oscillating pyruvate metabolism transcripts could contribute to the observed reduction in GNG output detected in SPF LKO and both GF groups (Figure 1D). KEGG “2-oxocarboxylic acid metabolism,” which includes pyruvate metabolism, also exhibited loss of enrichment in both LKO groups while WT groups exhibited enrichment (Figure 7).

In addition to glucose metabolism pathways, we also noted differential enrichment of lipid metabolism pathways (Figure 7). Reactome “Metabolism of lipids” was differentially enriched in all groups, where the SPF WT group exhibited the greatest enrichment, the SPF LKO group was intermediate, and both GF groups exhibited the lowest enrichment. This signifies a deviation from normal (SPF WT) oscillation patterns in transcripts that are key mediators of lipid metabolism. Importantly, several FA pathways, including “Fatty acid metabolism,” “Biosynthesis of unsaturated fatty acids,” several linoleic acid-related pathways, “Peroxisome,” and “PPAR signaling pathway” lacked any significant enrichment in SPF LKO oscillating transcripts, with reduced or absent enrichment across both GF groups. The recurrence of altered FA metabolic pathway enrichment, alongside altered pathways associated with GNG expression, supports our previous claim of a need for concurrent and synchronized regulation of these two metabolic pathways that impart key effects on global glucose regulation.

Together, diurnal oscillations of hepatic gene transcription are significantly altered in absence of a functional liver clock, gut microbes, or both. Although LKO and GF mice exhibited increased and unique oscillating transcripts, the normal enrichment of key GNG and FA metabolic pathways is reduced or lost in LKO and GF mice, further supporting the impaired efficiency of these metabolic processes in vivo.

### Gut microbes affect liver clock–driven network coexpression of transcripts.

**Figure 8:**
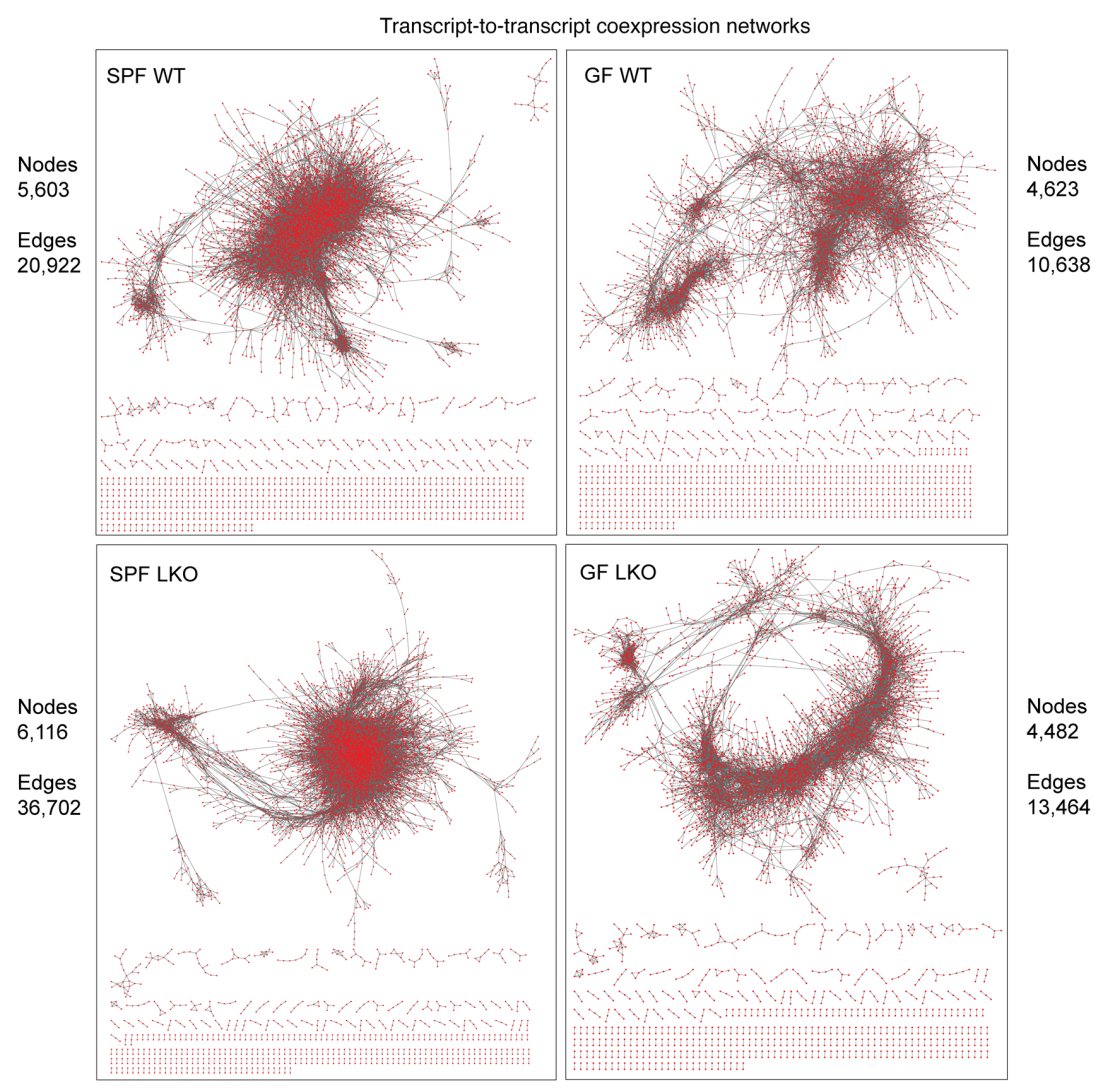
Hepatic transcriptome coexpression over time is differentially affected by the liver clock and gut microbes. Network transcriptome analysis of liver samples collected every 4 hours over 24 hours from SPF and GF WT and LKO male mice (*n* = 3/time point/group) maintained in 12:12 LD (ZT2, -6, -10, -14, -18, and -22). Network coexpression analysis of correlating transcripts over time within each group (*P* < 0.001). Network visualization and the number of correlating transcripts (nodes) and connections (edges) in each group. Red dots represent nodes, and gray lines represent edges.

We examined whether liver clock or gut microbes imposed an effect on transcript-to-transcript correlations over time using coexpression network analysis (Figure 4A, approach 3). We calculated Spearman’s correlation coefficients via pairwise-comparisons of transcript reads over time to identify significant co-occurrences for network visualization (Figure 8; network statistics in Supplemental Figure 8B). This allowed for the identification of nodes (correlated transcripts, *P* < 0.001) and their corresponding edges (connections between nodes). The SPF LKO group exhibited a modest increase in total nodes relative to the WT group (6,116 vs. 5,603); however, the number of edges increased from 20,922 (WT) to 36,702 (LKO), which was visually recognized by the density of the SPF LKO network compared with the WT network (Figure 8). Conversely, the total number of nodes and edges did not vastly differ between the GF WT and LKO groups. Additionally, the overall density of GF networks, regardless of liver clock status, was greatly reduced in comparison to SPF.

Next, KEGG annotations were applied to significantly correlated nodes and edges. While the number of nodes annotated to Carbohydrate (KO09101), Lipid (KO09103), and Amino acid (KO09105) metabolic pathways was not vastly different between groups, SPF LKO exhibited a 2-fold increase in edges annotated to these pathways compared with SPF WT (Table 1 and Supplemental Figure 8, C–E), while no difference was observed between GF groups. Despite a modest influence of liver clock or gut microbes on the total number of connected transcripts, loss of a functional liver clock, specifically in the presence of microbes, resulted in significant increases of abnormal connections between transcripts belonging to key metabolic pathways involved in GNG regulation. This demonstrates the combinatorial action between gut microbes and liver clock in the overall organization of hepatic metabolic gene transcription over time.

These data suggest both the liver clock and gut microbes aid in maintaining temporal coexpression of critical metabolic functional outputs of hepatic transcripts. Loss of either driver results in the emergence of abnormal connections, specifically those involved in carbohydrate and lipid metabolic pathways.

### Interactions between liver clock and gut microbes result in altered lipid versus carbohydrate fuel utilization.

Given that absence of a liver clock and gut microbes alters glucose and lipid metabolism, we interrogated how behavior, fuel utilization, and fuel switching were affected in vivo via indirect calorimetry over 4 days via the Promethion High-Definition Multiplexed Respirometry System. First, we detected no differences in basal metabolic rate regardless of *Bmal1* or microbial status (Supplemental Figure 9A). While we previously observed a small, but significant increase in food intake in male SPF LKO mice compared with WT mice (Supplemental Figure 1B, right), in the metabolic cage set-up, we observed no difference in overall food intake between genotypes (Supplemental Figure 9B). This discrepancy could be due to differences in cohort size, time scale, and method of measurement and food delivery. Plotting hourly food intake revealed that feeding onset was more robust in SPF LKO mice compared with WT mice, while no differences were evident in GF mice (Supplemental Figure 9C). The altered feeding bouts in SPF LKO mice could contribute to the observed increase in oscillating microbes over a 24-hour LD cycle, as shown in Figure 3A. Interestingly, ambulatory motion over the same period was not different in SPF mice; however, GF LKO mice exhibited increased total ambulatory motion relative to their WT counterparts (Supplemental Figure 9, D and E). This indicates the liver clock and microbes interact to influence feeding behavior, while shifts in ambulation due to a disrupted liver clock are exaggerated by a lack of gut microbes.

**Figure 9:**
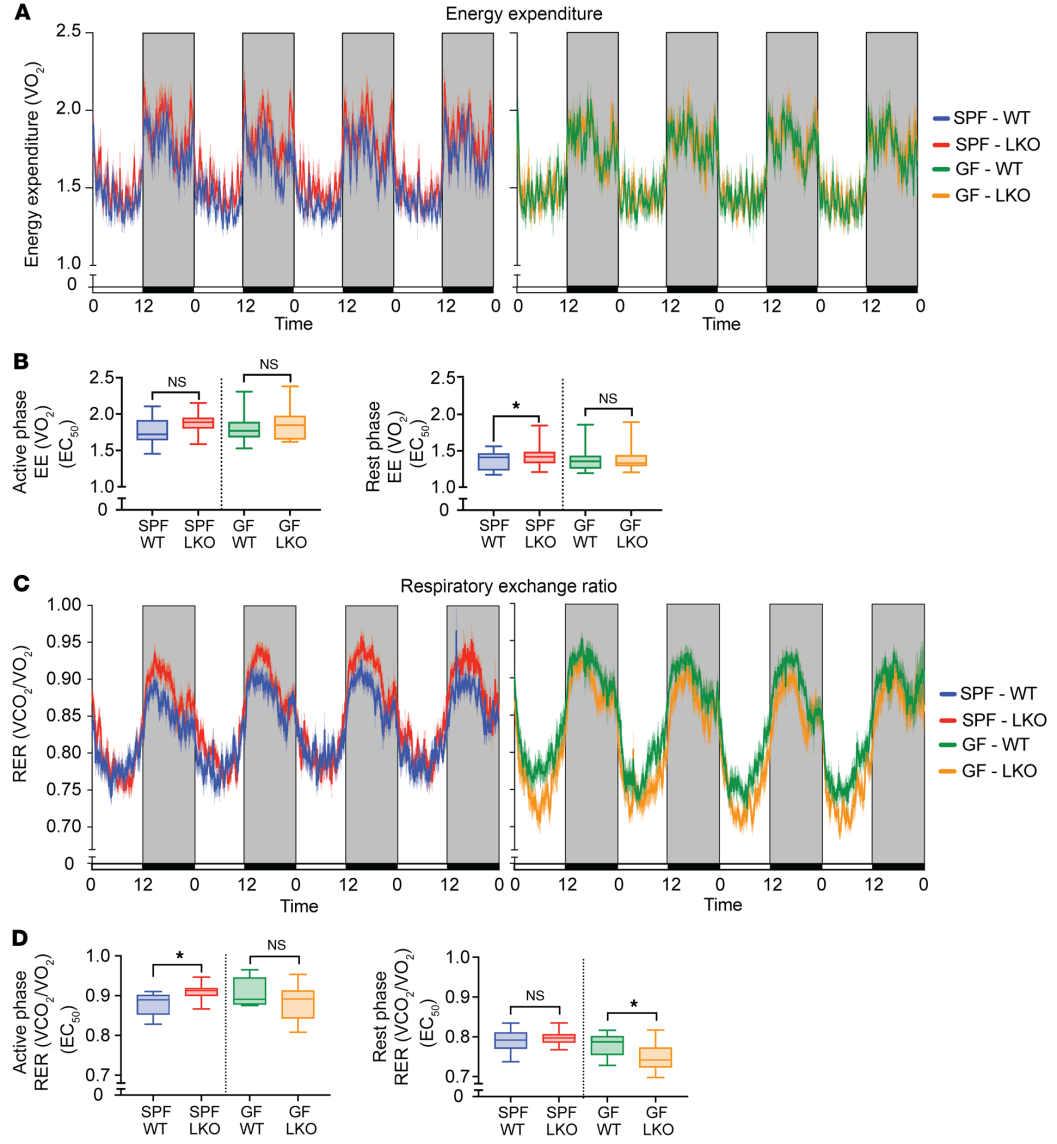
Liver clock and gut microbes differentially alter diurnal patterns of energy expenditure and fuel utilization. Indirect calorimetry assessment of SPF and GF WT and LKO male mice, measured over 4 consecutive 12:12 LD cycles (*n* = 12–13). (**A**) Energy expenditure (EE) represented as VO_2_. (**B**) EE divided into active (dark) and rest (light) periods, summarized by EC_50_ values within each period. (**C**) Respiratory exchange ratio (RER) represented as VCO_2_/VO_2_. (**D**) RER during active (dark) and rest (light) phases, summarized by EC_50_ values. Data points represent mean ± SEM. Lines in box plots represent the median, and whiskers represent the minimum and maximum, respectively. ANCOVA was performed between 2 groups. *P < 0.05.

We next examined energy expenditure via oxygen consumption (VO_2_) and fuel utilization via respiratory exchange ratio (RER; CO_2_ produced/O_2_ consumed). While no differences were detected between SPF genotypes during the active phase, we observed slight but significantly increased energy expenditure in SPF LKO mice compared with WT mice during the rest phase (Figure 9, A and B). These patterns were not evident in GF conditions. We then measured RER and found that SPF LKO mice exhibited significantly increased RER during the active period, implying a greater utilization of carbohydrates and reduced utilization of lipids (Figure 9, C and D). This suggests that a dysfunctional liver clock drives reduced utilization of lipids for fuel when microbes are present, supporting our evidence that GNG is also reduced. Conversely, no difference in RER was detected in GF mice regardless of genotype during the active phase, supporting our evidence that LKO mice exhibited reduced GNG compared with WT mice in SPF, but not in GF, conditions (Figure 1D). Interestingly, we observed decreased RER during the rest period only in GF LKO mice compared with GF WT mice, but not under SPF conditions (Figure 9, C and D), indicating that absence of both a liver clock and gut microbes may in fact enhance lipid oxidation relative to that observed in GF WT mice.

In summary, we found microbiota-dependent and -independent effects on liver clock–mediated fuel utilization. A reduced reliance on lipids in SPF LKO mice may contribute to reduced GNG and alter global metabolic homeostatic outputs.

## Discussion

**Figure 10:**
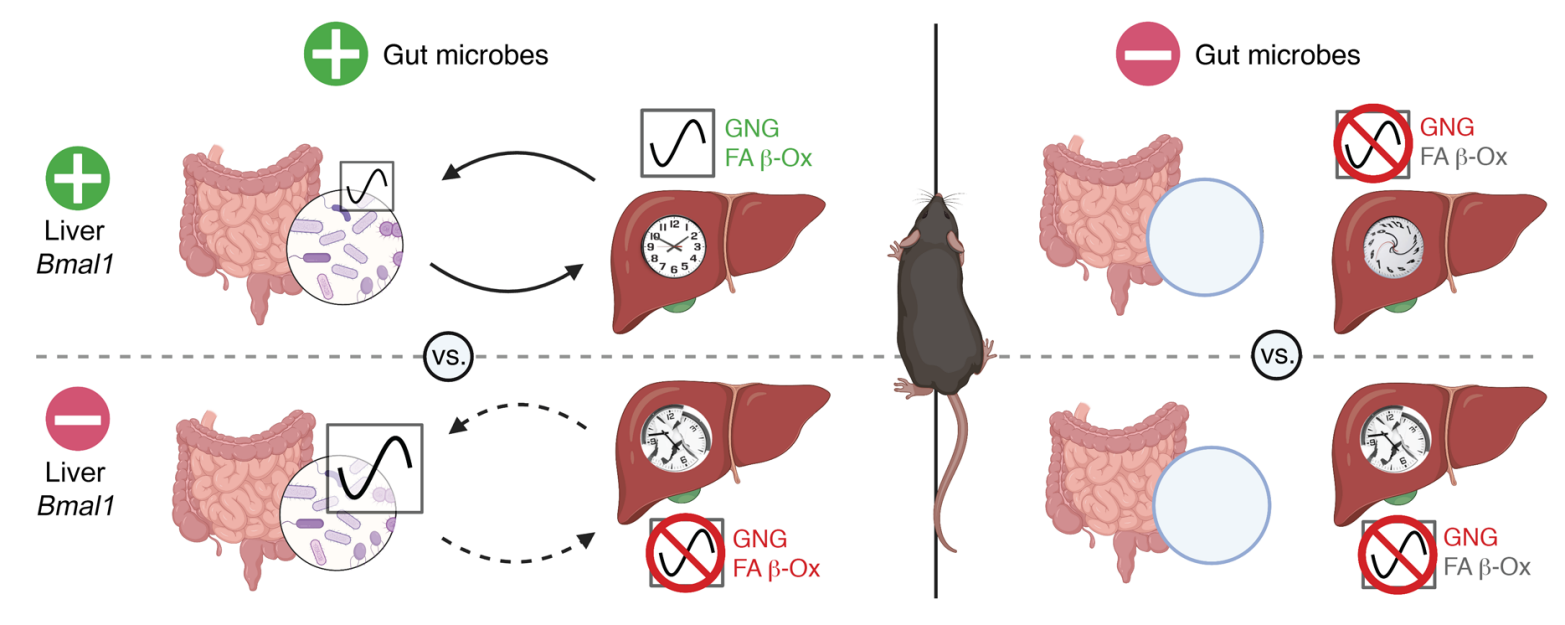
Liver clock and gut microbes partition glucose and lipid metabolism. Model figure. In SPF conditions, the liver circadian clock drives normal GNG and FA β-Ox, fecal microbial abundance oscillations, and hepatic transcriptome oscillations. Following hepatic *Bmal1* deletion, GNG and FA β-Ox are reduced, oscillating microbiota increase, and oscillating hepatic transcripts are not enriched for metabolic pathways, including GNG and FA β-Ox. In GF conditions, GNG is reduced, and oscillating hepatic transcripts are not enriched for GNG and FA β-Ox metabolic pathways regardless of liver clock functionality. Green indicates upregulation, and red indicates downregulation. Solid arrows indicate intact communication, and dashed arrows indicate broken communication. The figure created using BioRender.

Organismal-level coordination of molecular metabolism governed by the circadian clock is critical for the temporal separation of biochemical processes to maintain energy demands over a 24-hour period. Dysregulation or improper partitioning of these key processes can substantially contribute to metabolic diseases, such as obesity, nonalcoholic fatty liver disease, and type 2 diabetes. The liver is one of the most metabolically active organs, contributing to glucose and lipid homeostasis. Our data demonstrate a bidirectional and cooperative relationship between diurnal patterns of gut microbes and the liver circadian clock that aids in coordination of GNG, lipid metabolism, and fuel utilization, as outlined in Figure 10. This highly complex, reciprocal dialogue results in refined shifts that drive metabolic homeostasis. We found that the liver clock serves as the primary driver of diurnal transcriptional networks essential for maintenance of whole-body metabolism, whereas gut microbes are a secondary, essential partner that translates environmental cues (e.g., what, when, and how much diet is consumed) to enhance temporal organization of clock-mediated hepatic gene expression. When either system is absent or dysfunctional, disorganization and mistiming of normally coordinated processes occurs and homeostatic mechanisms are lost. These findings provide an initial basis for the interrogations of the mechanistic underpinnings of key host-microbe circadian interactions that direct metabolism in a tissue-specific manner.

A major finding of our study is that contributions of both gut microbes and the hepatic tissue-specific circadian network mediated glucose homeostatic outcomes, which occur independent of the central clock located within the brain (Figure 1, B and D and Supplemental Figure 1E). While others have separately implicated gut microbes (9, 22, 44) or the liver circadian clock (31) in the regulation of GNG and glucose tolerance, respectively, we showed that these systems work in conjunction to drive host glucose homeostasis. This appears to be a GNG-specific effect, supported by our evidence that both insulin sensitivity and hepatic glycogen levels were largely unaffected by LKO or GF conditions (Supplemental Figure 1, D and F). We found that gut microbes provide essential cues that mediate GNG, which can only be appropriately coordinated with the liver clock, a primary driver of maintaining metabolic partitioning. This engagement in microbiota-replete conditions underpins shifts between glucose and lipid metabolism for fuel utilization. By investigating these animals under GF conditions or following antibiotic-induced depletion of gut microbes in SPF mice (Figure 2), we were able to demonstrate that gut microbes contribute to these outcomes, serving in essence as a rheostat to impart signals in real time that fine-tune and modulate glucose and lipid metabolism. In the presence of gut microbes, gene-targeted deletion of the primary hierarchical driver, hepatic *Bmal1*, results in reduced host metabolic processes, like GNG, that are essential to maintain metabolic homeostasis. Altogether, these data underscore a key role for liver clock–microbiota crosstalk in maintaining circulating glucose levels, particularly during fasting conditions when GNG is critical. Whereas each system is greatly affected by environmental signals that influence metabolic outcomes, understanding their bidirectional dialogue is essential to comanipulate these systems in a meaningful and effective way.

In addition to differences in glucose homeostasis determined via direct measurements of GNG, we also identified that hepatic *Bmal1* and gut microbes coordinate diurnal patterns in lipid metabolism and subsequent fuel utilization via transcriptome analysis and indirect calorimetry, respectively (Figures 7 and 9). These findings, particularly in GF animals, provide insights into the role of liver *Bmal1* in these processes; previous work performed by our group and others in conventionally raised WT versus LKO mice showed the liver clock regulates lipid homeostasis via mechanisms involving AKT activation and m_6_A RNA methylation (37, 45). FA β-Ox imparts significant coregulation of GNG via several molecular signals, such as Acetyl-CoA, which is formed during fatty acid oxidation and allosterically activates pyruvate carboxylase, as well as cAMP, which induces both FA β-Ox and GNG via PPAR signaling (40, 46, 47). Thus, it is possible that our observations of liver clock–mediated GNG outputs under SPF and GF conditions may be a direct result of reduced hepatic FA β-Ox flux. Previous studies showed GF mice with a functional liver clock exhibit upregulation of FA β-Ox, even under high-fat diet–fed conditions (14); this could be due, in part, to differential regulation of hepatic PPAR signaling (48) and may provide protection against high-fat diet–induced obesity. Our studies in GF WT and LKO mice suggest that gut microbes also interact with the liver clock to regulate lipid metabolism, yet the precise microbial component(s) driving these outcomes remains to be determined. Given the relationship between FA β-Ox and GNG, we must consider the potential effects that high-fat diet may have on the individual outputs of these pathways and their coregulation. For instance, a previous study revealed that high-fat dietary intake in *Bmal1* liver–deficient mice resulted in increased body weight gain and disrupted mitochondria functional outputs compared with WT counterparts (49). We suspect that exposure to high-fat diet would impact the liver clock–microbiome relationship, perhaps exacerbating the lipid and associated GNG outputs that we observe under grain-based, undefined chow-fed conditions in SPF mice. This will be a focus of future studies for our group.

In addition to the metabolic implications of gut microbes for the host, we demonstrate bidirectionality from the host liver clock to gut microbes. Here, functionality of the liver clock affects diurnal oscillations of fecal microbiota relative abundances. Loss of a primary hepatic driver, such as *Bmal1*, results in the emergence of downstream secondary microbiota oscillators that could directly feedback onto the host, disrupting proper feedback mechanisms. Previously, Liang et al. (26) showed that global loss of *Bmal1* in mice abolished fecal microbial abundance rhythms compared with those in WT mice. In contrast, our work shows that absence of tissue-specific *Bmal1* resulted in an almost 2-fold increase in oscillating ASVs, particularly within the families Lachnospiraceae and Ruminococcaceae (Figure 3), which we further confirmed by log-ratio analysis (Supplemental Figure 6). This indicates that the tissue-specific liver clock is a key player in maintaining normal rhythmicity of specific oscillating gut microbiota. Others have shown that these bacteria families positively correlate with and contribute to secondary bile acid metabolism (50, 51), as well as circadian rhythm regulation and metabolism (52, 53). Furthermore, previous studies have revealed that PPAR signaling is linked to bile acid synthesis and regulation (54, 55). In fact, we observed reduced levels of transcripts involved in PPAR signaling in hepatic *Bmal1*-deficient mice. Whether the observed gain in oscillating ASVs in LKO SPF mice contributes to altered circulating bile acid pools that impact glucose metabolism remains to be investigated. Gains in oscillation have been previously identified in the context of microbiota-relative abundances and associated metabolites in metabolic disease (23, 56), yet their precise meaning remains unclear. Importantly, not only did we observe a gain in oscillations of gut microbiota, but we also found an increase in oscillations of the host liver transcriptome in absence of either a functional liver clock or gut microbes (Figure 6A). These gains of rhythmicity confer differential enrichment of key metabolic pathways compared with SPF WT mice (Figure 7). This finding corresponds to previous observations that the emergence of unique oscillations can significantly alter functional transcriptome enrichment that affects metabolic homeostasis (42, 57).

Our study underscores that gut microbiota play a key role in mammalian metabolic homeostasis mediated through interactions and coordination with primary drivers of hepatic circadian networks. Molecular signals derived from the gut microbiome that impart liver clock–mediated GNG and overall metabolic organization remain undefined, and their identification will be an essential next step to gain further insights. In preliminary investigations, we examined short-chain fatty acids (SCFAs) as a potential mediator of this interaction; however, absence of a liver clock did not appear to alter either SCFA levels or diurnal dynamics in SPF WT versus LKO mice (data not shown). Another mediator candidate that may mechanistically link these systems is glucagon-like peptide 1 (GLP-1), an incretin produced by intestinal L cells that induces insulin secretion and glucose uptake. Not only does GLP-1 exhibit robust diurnal patterns that are required for normal glucose homeostasis, but its secretion is also significantly induced by loss of gut microbes (22, 58); additional experiments will be required to investigate this thoroughly. Due to the complex nature of host-microbe interactions, it is possible that a single mediator is not responsible; instead there is a possibility that a combination of factors contributes to the mutual and temporal regulation of these systems. Future studies aimed at understanding how these microbial mediators interact with circadian networks, particularly in humans, will be an important step in the field. Together, our physiological and multi-omic data highlight key communication between the host hepatic circadian clock and gut microbiota, underscoring the importance of proper diurnal coordination of these two systems. These findings could have broader translational implications for how these two systems might be targeted to improve metabolic homeostasis in humans.

## Methods

Specific catalog numbers for antibodies, animals, and reagents are listed in Supplemental Table 2.

### Animals.

All mice used in these studies were on a C57BL/6J background. SPF *Bmal1^fl/fl^* and Albumin-Cre male and female mice were purchased from The Jackson Laboratory and bred in The University of Chicago vivarium as previously described (31). SPF *Bmal1^fl/fl^* Alb-Cre mice were rederived under GF conditions and maintained in flexible film isolators (CBC Ltd.) in The University of Chicago Gnotobiotic Research Animal Facility. To achieve forebrain-specific knockout of *Bmal1*, SPF *Bmal1^fl/fl^* mice were crossed with *CamiCre* mice, which were a gift from Joseph Takahashi with the Department of Neuroscience and the Howard Hughes Medical Institute at University of Texas Southwestern Medical Center (33). All mice were maintained under 12:12 light/dark conditions (lights on at 6 am, ZT0) and fed autoclaved ad libitum JL Rat and Mouse/Auto 6F 5k67 (LabDiet) for at least 2 weeks prior to and throughout experiments. Body weight and food consumption were monitored weekly. At 10–14 weeks of age, mice were acclimatized to individual housing for 14 days, after which stool was collected every 6 hours for 48 hours. After 3 weeks, mice were sacrificed via CO_2_ asphyxiation followed by cervical dislocation over a 24-hour period at 6 time points: ZT2 = 8 am, ZT6 = 12 pm, ZT10 = 4 pm, ZT14 = 8 pm, ZT18 = 12 am, and ZT22 = 4 am. Immediately prior to sacrifice, basal blood glucose was measured from tail snip blood via Accu-Check Compact Plus Diabetes Monitoring Kit and test strips (Roche Diagnostics) or OneTouch Ultra 2 Blood Glucose Monitoring System (LifeScan). Blood was collected via heparin-coated microvette tubes (Sarstedt) for insulin measurement by the Ultra-sensitive Insulin ELISA (ALPCO). Liver, plasma, brain, and intestinal luminal contents were snap frozen in liquid nitrogen and stored at –80°C.

### Conventionalization studies.

Fresh stool from adult C57BL/6J male mice was resuspended in sterile PBS (100 mg stool/mL), vortexed, and spun briefly to remove debris. Individually housed GF *Bmal1^fl/fl^* or *Bmal1^fl/fl^-AlbCre* 11- to 13-week-old ad libitum–fed male mice were orally gavaged with 150 μL suspension. Body weights and food consumption were monitored weekly.

### Antibiotic treatment studies.

For antibiotic treatment, SPF *Bmal1^fl/fl^* or *Bmal1^fl/fl^-AlbCre* 11- to 13-week-old male mice were administered an antibiotic cocktail consisting of vancomycin (0.5 mg/mL), neomycin (1 mg/mL), and cefoperazone (0.5 mg/mL) prepared in autoclaved water and sterile filtered through a 0.2 μM filter. The protocol involved using a combination of daily gavage for 7 days followed by incorporation and ad libitum delivery in drinking water for 1 additional week. Body weights were monitored daily.

### Primary hepatocyte isolation and culture.

Isolation and culture of primary hepatocytes were performed as described in Mouse Liver Cells (http://mouselivercells.com). 15-week-old SPF and GF *Bmal1^fl/fl^* and *Bmal1^fl/fl^-AlbCre* male mice were anesthetized (90 mg ketamine/kg body weight, 6 mg xylazine/kg body weight), perfused via portal vein cannulation with Collagenase Type IV (Worthington Biochemical) in situ, digested in low-glucose DMEM (Corning), and isolated in high-glucose DMEM (Gibco). Live hepatocytes were seeded at 600,000 hepatocytes in 6-well collagen-coated plates (Thermo Fisher Scientific) and cultured in low-glucose DMEM (Corning) for 2 days with daily media change prior to assay.

### Oral glucose, intraperitoneal pyruvate, and intraperitoneal ITTs.

For GTT and PTT, 12- to 16-week-old male GF and SPF mice were fasted overnight for 14 hours, starting at ZT12. For ITT, mice were fasted for 5 hours, starting at ZT22. Baseline blood glucose was measured via tail snip using a hand-held glucometer. Mice were administered an oral bolus of 20% dextrose (Hospira) in sterile water solution (2 g/kg body weight), an intraperitoneal injection of sterile-filtered sodium pyruvate (Sigma-Aldrich) in PBS (2 g/kg body weight), or an intraperitoneal injection of 0.1 U/mL Humulin R Insulin (Eli Lilly) in PBS (1 U/kg body weight). For GTT and PTT, blood glucose was measured at 1, 30, 60, and 120 minutes after gavage or injection. For ITT, blood glucose was measured at 15, 30, 60, 90, and 120 minutes after injection. During GTT, insulin levels were determined at baseline, 30, 60, and 120 minutes in blood collected in heparin-coated microvette tubes (Sarstedt) using the Ultra-sensitive Insulin ELISA (ALPCO). AUC was calculated using GraphPad Prism v9.

### Western blot.

Liver and brain tissue (5–10 mg) were collected at ZT16 and placed in 250–500 μL ice-cold RIPA lysis buffer (Thermo Fisher Scientific RIPA Lysis and Extraction Buffer, cOmplete Mini Protease Inhibitor Cocktail, 100 μM PMSF). Protein concentrations were determined via Bicinchoninic Acid Protein Assay (Thermo Fisher Scientific), and 30 μg protein was separated on 4%–20% Mini-PROTEAN gel (Bio-Rad) and transferred to a PVDF membrane (Millipore). Membranes were blocked with 5% nonfat milk in Tris-buffered saline (TBS) (20 mM Tris pH7.6, 150 mM NaCl) and incubated overnight at 4°C in 5% nonfat milk in TBS-Tween (TBS-T) (20 mM Tris pH7.6, 150 mM NaCl, 0.1% Tween-20) containing primary antibodies: anti-BMAL1 (1:1,000; Abcam, ab93806) or anti-GAPDH (1:100,000; Invitrogen, AM4300). Membranes were washed 6 times for 5 minutes in TBS-T and incubated for 1 hour at room temperature in TBS-T containing 5% nonfat milk and either anti-mouse or anti-rabbit secondary antibody (1:10,000; Abcam, 7076S and 7074S). Membranes were washed again, developed with SuperSignal West Pico PLUS Chemiluminescent Substrate (Thermo Fisher Scientific), and exposed using the Chemi-Doc MP Imaging System (Bio-Rad Laboratories).

### Primary hepatocyte glucose production assay.

Cultured mouse hepatocytes were serum-starved overnight and washed with PBS with MgCl_2_ and CaCl_2_ (Sigma-Aldrich). Hepatocytes were exposed to glucose-free DMEM (Sigma-Aldrich) (20 mM sodium lactate, 2 mM sodium pyruvate, 2 mM L-glutamine, 15 mM HEPES) with or without 0.1 mM pCPT-cAMP (Sigma-Aldrich) for 11 hours. Media was collected and glucose concentrations were measured via Autokit Glucose enzymatic assay (FUJIFILM Medical Systems Inc.) and normalized to protein content determined via Bicinchoninic Acid assay.

### Glycogen assay.

Frozen liver samples were weighed, and glycogen measurement was performed using the Glycogen Assay Kit II (Colorimetric) (Abcam) following the manufacturer’s instructions.

### Bacterial gene quantification.

16S rRNA gene copy number was determined in stool as previously described (23, 59, 60). The 16S rRNA gene was quantified using a standard curve for gene copy number using primer sequences cloned into a PCR4-TOPO plasmid (see Supplemental Table 2, for primer sequences).

### 16S DNA extraction, sequencing, and analysis.

Stool was bead beaten using 0.1 mm diameter zirconia/silica beads (BioSpec Products) as previously described (23). Supernatants were extracted using equal volumes of phenol:chloroform:isoamylalcohol (25:24:1; Ambion), and DNA was precipitated using an equal volume of 100% isopropanol. DNA concentration was measured using the Quant-iT PicoGreen dsDNA Assay kit (Invitrogen) and diluted to 1–20 ng/μL. The V4–V5 region of the 16S rRNA encoding gene was amplified using standard Earth Microbiome Project protocols. Sequencing was performed at the High-Throughput Genome Analysis Core (part of the Institute for Genomics and Systems Biology, Argonne National Laboratory [Lemont, Illinois, USA]). Paired-end reads were imported into Quantitative Insights Into Microbial Ecology version 2 (QIIME2) software suite (61) (https://qiime2.org) and demultiplexed using Dada2 (62). Filtering steps were performed, including filtering out samples containing less than 1.5 times the standard deviation from the mean of sample read counts across all samples within the experiment and filtering out features that appear in less than 10 samples across the experiment. To maximize sampling depth while prioritizing equal retention of samples across groups, 10,598 (Figure 3 and Supplemental Figures 4 and 5; 48-hour fecal collection) or 15,578 (Supplemental Figure 3; cohousing vs. separate housing fecal collection) reads were included per sample, determined by the mean subtracted by 1.5 times standard deviation of sample read counts across all samples within the experiment. Taxonomy was compiled using the classify-sklearn plugin with Silva database version 132 2020.8 (63, 64). β-Diversity analyses and permutational multivariate ANOVA statistics were performed via QIIME2.

### Liver RNA extraction, sequencing, and analysis.

Total RNA was isolated by homogenizing bulk liver tissue in TRIzol (Ambion) followed by chloroform extraction, as previously described (23). RNA quality and quantity were assessed using the Agilent bioanalyzer, and RNA-SEQ libraries were generated using the Illumina Stranded mRNA Prep at The University of Chicago Genomics Core Facility. Sequencing of mRNA directional, single-end 50 base pair reads was performed on the HiSEQ4000 with Illumina reagents and protocols. Data was demultiplexed using the Illumina bcl2fastq software. All 72 samples were run on 6 lanes, and fastq files were concatenated. Quality control was performed using FastQC v0.11.5 and MultiQC v1.10.1. Sequence alignment was performed by STAR version 2.6.1b by mapping to the mm10 whole genome (65). Gene transcript counts were determined by Subread:featurecounts v2.0.0.

Differential analysis was performed via DESeq2, both on all time points pooled and within each individual time point of variance-stabilizing transformation (VST) data. Protein coding genes were identified via biomaRt (version 2.40.5) using GRCm39 mouse genes. Two-way analysis for each pair of experimental groups were performed. The Wald statistic, calculated via DESeq2, was used to build a ranked list of genes for each comparison for each time point. fGSEA (version 1.10.1) was utilized to identify differentially regulated pathways (with parameter nperm = 10,000) within multiple annotated gene sets from Molecular Signatures Database (MSigDB, version 7.0; https://www.gsea-msigdb.org/gsea/msigdb). Significantly identified pathways were filtered by adjusted *P* < 0.05 and subsequently binned into categories via custom R script. fGSEA was utilized to calculated normalized enrichment score and identify leading edge genes.

For oscillation analysis, feature counts were normalized by VST in DESeq2 (version 1.24.0). VST-normalized data were used for principal component analysis. VST-normalized data were analyzed to identify significantly oscillating transcripts via empirical JTK_CYCLE (66). Heatmaps were generated from VST-normalized data, ordered by time of peak expression, normalized by the median value for each transcript within each group over time, and visualized using Orange3 (https://orangedatamining.com). Metascape was utilized to identify statistically enriched pathways from each list of oscillating transcripts (43) (http://metascape.org), which were then binned into categories via a custom R script.

Network coexpression analysis was performed using RNA-Seq raw transcript abundance counts. Transcripts with low abundance counts (<50 counts in all samples or genes with 0 counts in more than 2 samples per time point) were removed. Spearman’s correlation values for comparisons between pairs of transcripts were calculated within each genotype. Transcript count values were randomly selected from 1 of the 3 samples for a given time point, and then correlation *P* and *r* values between transcripts over time were calculated in R and repeated over 500 permutations (*‘dplyr’, ‘rcorr’, ‘Hmisc’*). Correlation matrices for the permutations were averaged to generate a single matrix of transcript correlation values. The matrix was flattened into a network-importable table format in R (*‘cormat’, ‘pmat’*): flatCorrMatrix < -function(cormat,pmat){ut < -upper.tri(cormat)data.frame(row = rownames(cormat)[row(cormat)[ut]],column = rownames(cormat)[col(cormat)[ut]],cor = (cormat)[ut],p = pmat[ut]). Correlations were filtered for *P* < 0.001 and Spearman’s *r* > 0.95, and all self-relationships removed. This filtered correlation table was used for network visualization in Cytoscape (67) (https://cytoscape.org) as well as for downstream analyses of node connectivity and centrality. Annotations from the KEGG were added (https://www.ensembl.org) and utilized to identify transcript nodes in each network with specific functionally related pathways.

### Metabolic cage studies.

Mice were acclimatized to individual housing for 5 days prior to metabolic monitoring, which was conducted using the Promethion High-Definition Multiplexed Respirometry System for Mice (Sable Systems International). All measurements were recorded at 3-minute intervals across all cages. Food and water intake were recorded by gravimetric measurement. Physical movement was determined by infrared sensor beam breaks. Oxygen consumption, carbon dioxide production, and respiratory quotient were measured by indirect calorimetry. Energy expenditure (kcal/h) was calculated by the Weir equation (68).

Basal metabolic rate was measured on the first day of metabolic cage housing. Following transfer into metabolic cages at ZT3, food was immediately removed. Between ZT7 and ZT10, the lowest average postabsorptive energy expenditure value over a 5-minute period was identified as BMR (69). Food was returned at ZT10, mice were allowed to acclimatize for 2 days, followed by 4 days of resting metabolic rate measurements. For GF mice, validation of GF status was performed using 16S rRNA gene PCR on DNA extracted from fecal samples collected before and after metabolic caging.

All data were recorded via IM3 software v21.0.2 and converted to XML via a custom Sable Systems macro. Data were cleaned in Python, including identification of each diurnal cycle using environmental lux values. Within each period, sum was calculated for beam breaks and movement, and range was calculated for food and water intake. EC_50_ (70) was calculated for RER, VO_2_, and energy expenditure in R using nplr v.1-7. Analysis of covariance (ANCOVA) (body weight as covariant) was performed for each measurement between 2 groups using R.

### Statistics.

Data are presented as mean ± SEM or box-and-whisker plots showing the median ± minimum/maximum. Statistical analysis was performed using either GraphPad Prism v9 software or R packages. Two-tailed unpaired Welch’s *t* tests or ANCOVA were performed between 2 groups, and Brown-Forsythe and Welch’s 1-way ANOVA followed by Dunnett’s tests were performed among 3 or more groups. *P* < 0.05 was considered statistically significant. Metascape was used to identify significantly enriched pathways from oscillation transcriptome profiles with *q* < 0.05. Spearman’s correlation was performed to identify significantly correlated gene expression over time with *P* < 0.001 and *r* < 0.95. fGSEA was used to identify significantly enriched pathways from differential gene expression analysis with adjusted *P* (padj) < 0.05 and nperm = 10,000. CircWave V1.4 (http://clocktool.org) or empirical JTK_CYCLE (66) were used to identify significantly oscillating data by *P* < 0.05 (CircWave) or GammaBH < 0.05 (*P* value was calculated from gamma fit of empirical null distribution, adjusted for Benjamini-Hochberg false discovery rate, via eJTK). Log-ratio analyses for 16S data were performed via Songbird using methodology outlined in Morton et al. (36). In addition to default parameters, the equation was set to “C(Time, Treatment(‘Two’), pmin of feature counts set to 0, epochs set to 40,000, learning rate set to 0.0001, and trainer samples set to 3. The resulting log ratios were sorted hierarchically in a heatmap, plotted over time via LOESS fitting, and fitted for nonzero base sinusoidal fits. Statistical outliers were identified as 2 standard deviations ± mean and removed from the analysis. Metabolic cage statistical outliers were removed based on S_n_ values (71) using the R package robustbase (https://robustbase.r-forge.r-project.org). A consistency factor of 1.1926 was used to calculate the S_n_ value for each channel within each cycle, and outliers were defined as ± 3 times the S_n_ value from the median. Python v3.7.5 and R v3.6.3 were used.

### Study approval.

All animal protocols and experimental procedures were approved by The University of Chicago Institutional Animal Care and Use Committee.

### Data availability.

16S rRNA sequences are available for download at the NCBI Sequence Read Archive (accession PRJNA815335). RNA sequences are available for download at the NCBI Gene Expression Omnibus (accession GSE184303).

## Author contributions

This manuscript was conceptualized by KF, VAL, and EBC. Data analysis was performed by KF, S Manzoor, OD, KC, EAC, and VAL. Experiments were conducted by KF, S Manzoor, KC, S Miyoshi, JM, MS, AT, and VAL. Reagents were provided by MI and JST. Manuscript writing, review, and editing was performed by KF, MCR, VAL, and EBC. Funding was acquired by KF, MCR, VAL, and EBC.

## Supplementary Material

Supplemental data

Supporting data values

## Figures and Tables

**Table 1:**
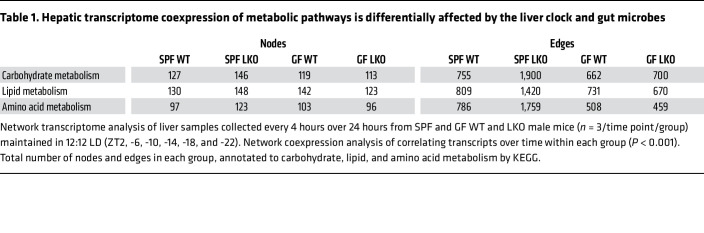
Hepatic transcriptome coexpression of metabolic pathways is differentially affected by the liver clock and gut microbes

## Version 1 09/15/2023

Electronic publication
