# Supplementary material for: Gut microbes and the liver circadian clock partition glucose and lipid metabolism
Source: J Clin Invest. 2023 Sep 15;133(18):e162515. doi: 10.1172/JCI162515 (PMC10503806; doi:10.1172/JCI162515)
Supplement: Supplemental data [file jci-133-162515-s233.pdf]

**Supplemental Data**

# Gut microbes and the liver circadian clock partition glucose and lipid metabolism

Katya Frazier[1,2], Sumeed Manzoor[1], Katherine Carroll[1], Orlando DeLeon[1], Sawako Miyoshi[3], Jun Miyoshi[4], Marissa St George[1], Alan Tan[1], Evan A. Chrisler[5], Mariko Izumo[6], Joseph S. Takahashi[6,7], Mrinalini C. Rao[1], Vanessa A. Leone[5#], & Eugene B. Chang[1,2#]

**Supplemental Figure 1, related to Figure 1. The liver circadian clock and gut microbes do not mediate insulin tolerance, hepatic glycogen, or *ex vivo* hepatic GNG.**

**(A)** Western blot of BMAL1 and GAPDH (control) in brain (frontal lobe, hypothalamus, cerebellum) and liver protein extracts collected at ZT16 from male *Bmal1*$^{f/f}$(WT), *Bmal1*$^{f/f}$ *Alb-Cre* (LKO), and *Bmal1*$^{f/f}$ *Cam-Cre* (Forebrain-*Bmal1*-KO). **(B)** Body weight (left panel) and weekly food intake (right panel) from SPF and GF, WT and LKO male mice (*n*=18-32/group). **(C)** Liver (left panel) and mesenteric & gonadal (right panel) fat weight from SPF and GF, WT and LKO male mice, presented as percent of body weight (*n*=27-34/group). **(D)** Intraperitoneal Insulin Tolerance Test in SPF and GF, WT and LKO male mice (ITT, n=11-17/group). **(E)** PTT in SPF WT and Forebrain-*Bmal1*-KO male mice (*n*=11-16/group). **(F)** Glycogen content in liver tissue from SPF and GF WT vs LKO male mice collected every 4 hrs over 24 hrs from animals maintained in 12:12 LD (*n*=5-7/group); CircWave statistics indicate significantly oscillating ($p<0.05$) or not oscillating ($p>0.05$) values. **(G)** Glucose production by primary hepatocytes, isolated from SPF and GF WT vs LKO male mice, following 11 hrs of treatment with vehicle control (Basal) vs pCPT-cAMP, normalized to protein content and represented as arbitrary units (AU) (*n*=5/group). Data points represent mean±SEM, box plots presented as median±min/max. Two-tailed unpaired Welch's t tests were performed between 2 groups; Brown-Forsythe & Welch ANOVA followed by Dunnett's tests were performed between three or more groups; ****$p<0.0001$, ***$p<0.001$, **$p<0.01$, *$p<0.05$, ns=not significant; colored stars represent significance relative to SPF WT. Inset graph represents AUC normalized to baseline glucose values.

# Supplemental Figure 1

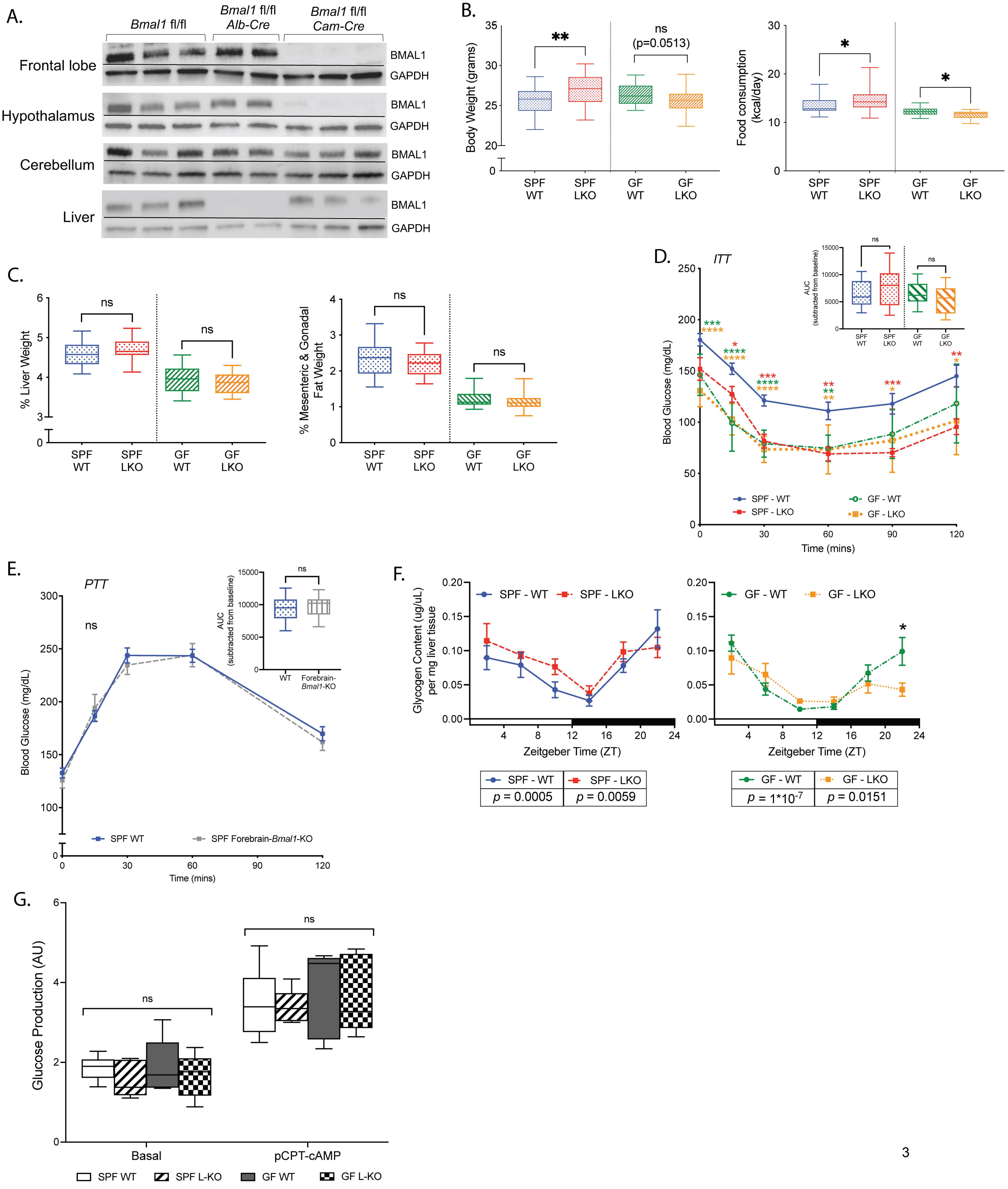

**Supplemental Figure 2, related to Figure 1. The liver circadian clock and gut microbes do not mediate glucose metabolism in female mice.**

**(A)** Body weight (left panel) and weekly food intake (right panel) from SPF and GF, WT and LKO male mice (*n*=26-34/group). **(B)** Resting blood glucose levels measured via tail snip of SPF and GF, WT and LKO female mice every 4 hrs over 24 hrs (*n*=5-7/group/timepoint, SPF and GF groups also shown separately). CircWave statistics indicate significantly oscillating ($p<0.05$) or not oscillating ($p>0.05$) values. **(C)** GTT in SPF and GF, WT and LKO female mice (*n*=8-10/group). **(D)** PTT in SPF WT and LKO female mice (*n*=13-14/group). Data points represent mean±SEM, box plots presented as median±min/max. Two-tailed unpaired Welch's t tests were performed between 2 groups; Brown-Forsythe & Welch ANOVA followed by Dunnett's tests were performed between three or more groups; **$p<0.01$, *$p<0.05$, ns=not significant; colored stars represent significance relative to SPF WT. Inset graph represents AUC normalized to baseline glucose values.

# Supplemental Figure 2

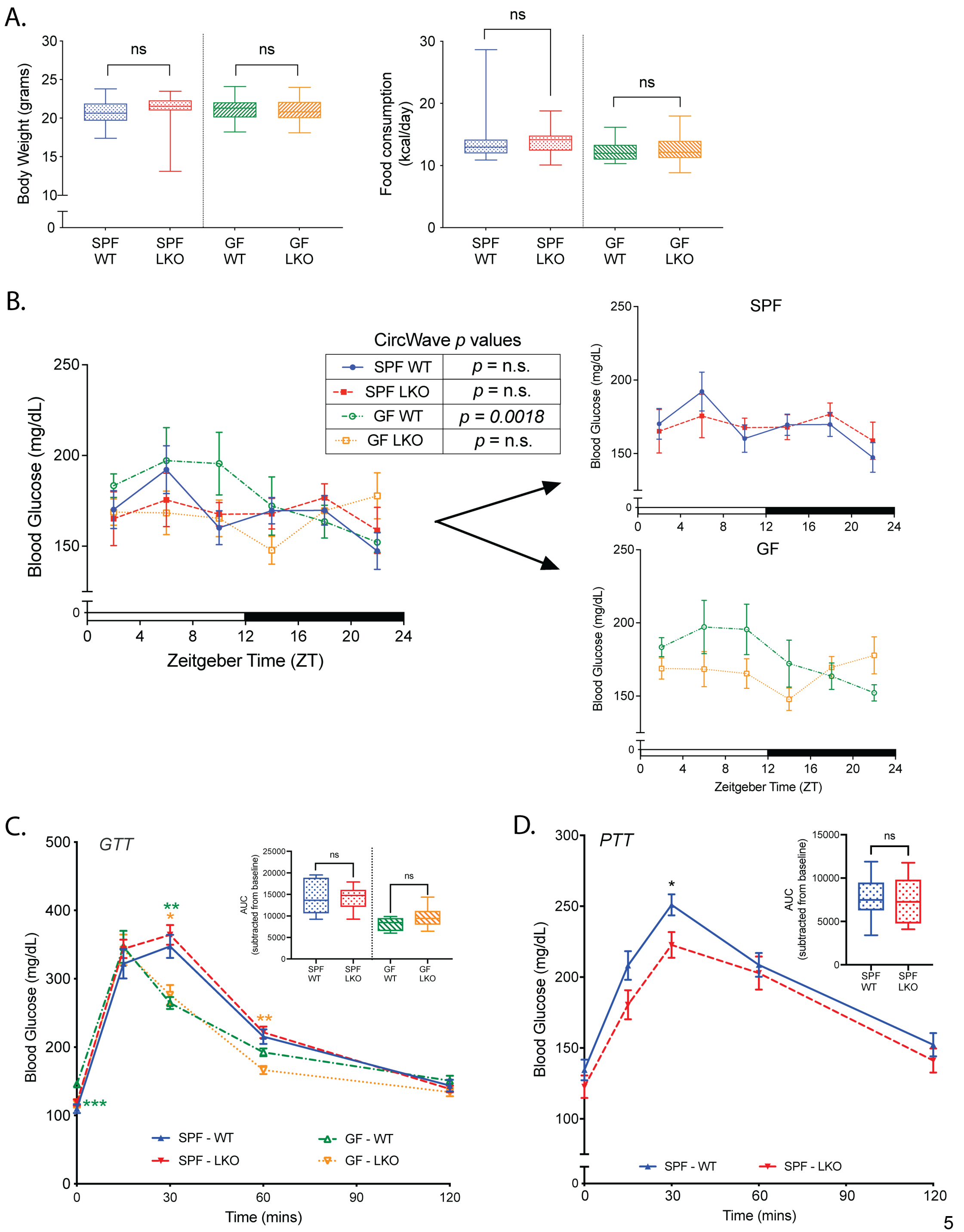

**Supplemental Figure 3, related to Figure 2. Liver circadian clock status does not impact gut microbial community membership.**

**(A)** 16S rRNA gene abundance in stool collected before (Pre-Abx) and after (Post-Abx) antibiotic treatment, measured via quantitative PCR and normalized to DNA concentration. **(B)** Experimental scheme of mixed vs. separate housing at time of weaning. **(C,D)** 16S rRNA gene sequencing analysis of stool collected from WT vs LKO male mice housed either by mixed genotypes (WT+LKO) or via separate housing of genotypes (WT+WT and LKO+LKO) (*n*=6-7/group); abundance counts of major **(C)** and minor **(D)** phyla. **(E)** Principal Coordinate Analysis (PCoA) of Bray-Curtis and Weighted Unifrac beta diversity analyses, with PERMANOVA q value statistics. Box plots represent median±min/max. Two-tailed unpaired Welch's t tests were performed between 2 groups; ns=not significant.

# Supplemental Figure 3

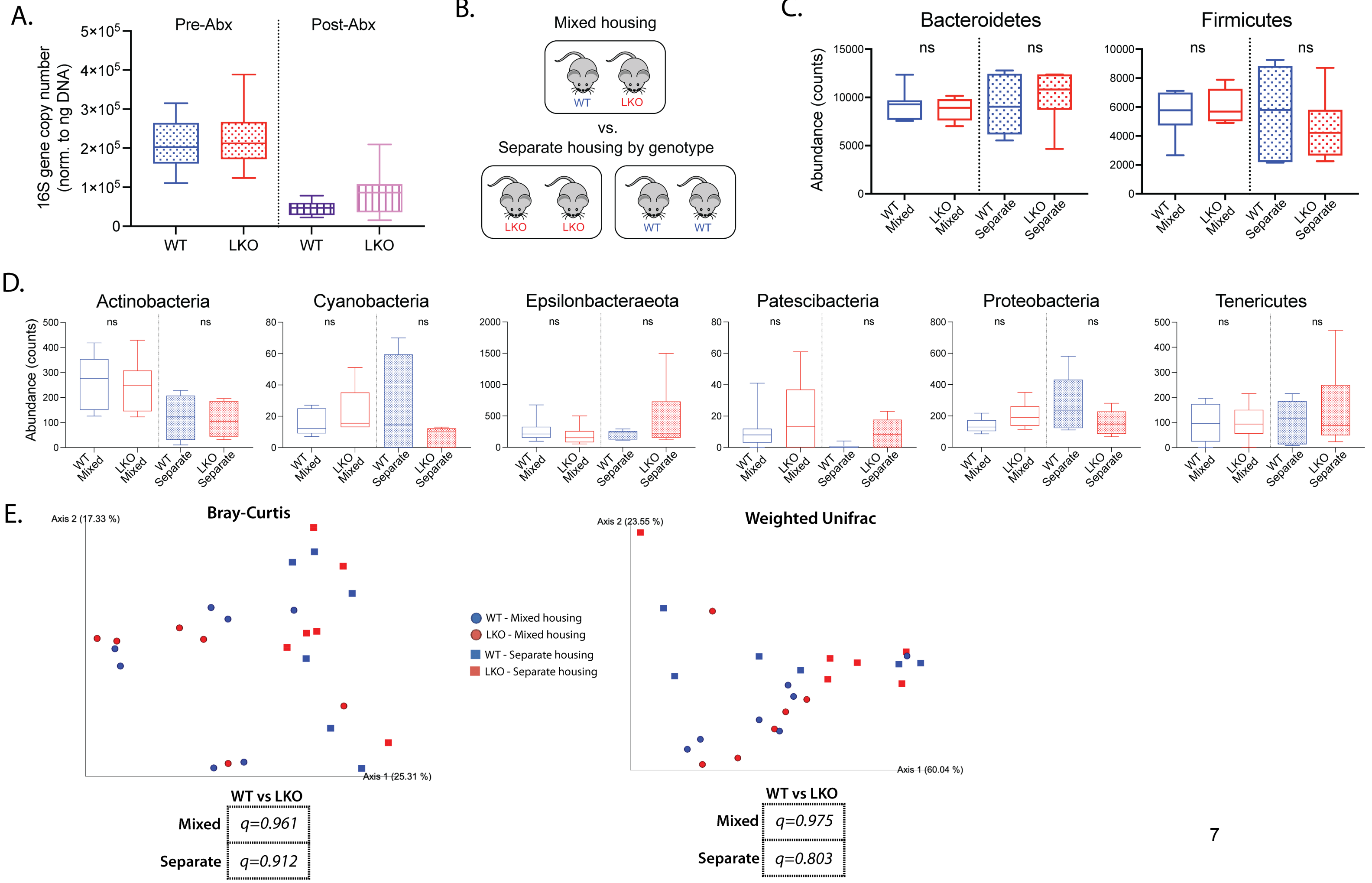

**Supplemental Figure 4, related to Figure 3. Loss of a functional liver clock in male mice results in increased abundance of oscillating ASVs.**

16S rRNA gene sequencing analysis of stool sampled from WT and LKO male mice every 6 hrs over two consecutive 24-hr LD cycles (8 samples per animal) via repeat collections from the same mouse (*n*=7-8/group). **(A)** Experimental scheme of 48-hr stool collection. **(B)** 16S rRNA gene abundance measured via qPCR normalized to DNA concentration. **(C)** Venn diagrams presenting total, shared, and unique oscillating ASVs among all taxonomic groups, as well as within specific classes Bacteroidales and Clostridiales. **(D)** Abundance counts of all oscillating ASVs. **(E)** Abundance counts of total vs oscillating ASVs within *Lactobacillus murinus*. Data points represent mean±SEM. ns=not significant.

# Supplemental Figure 4

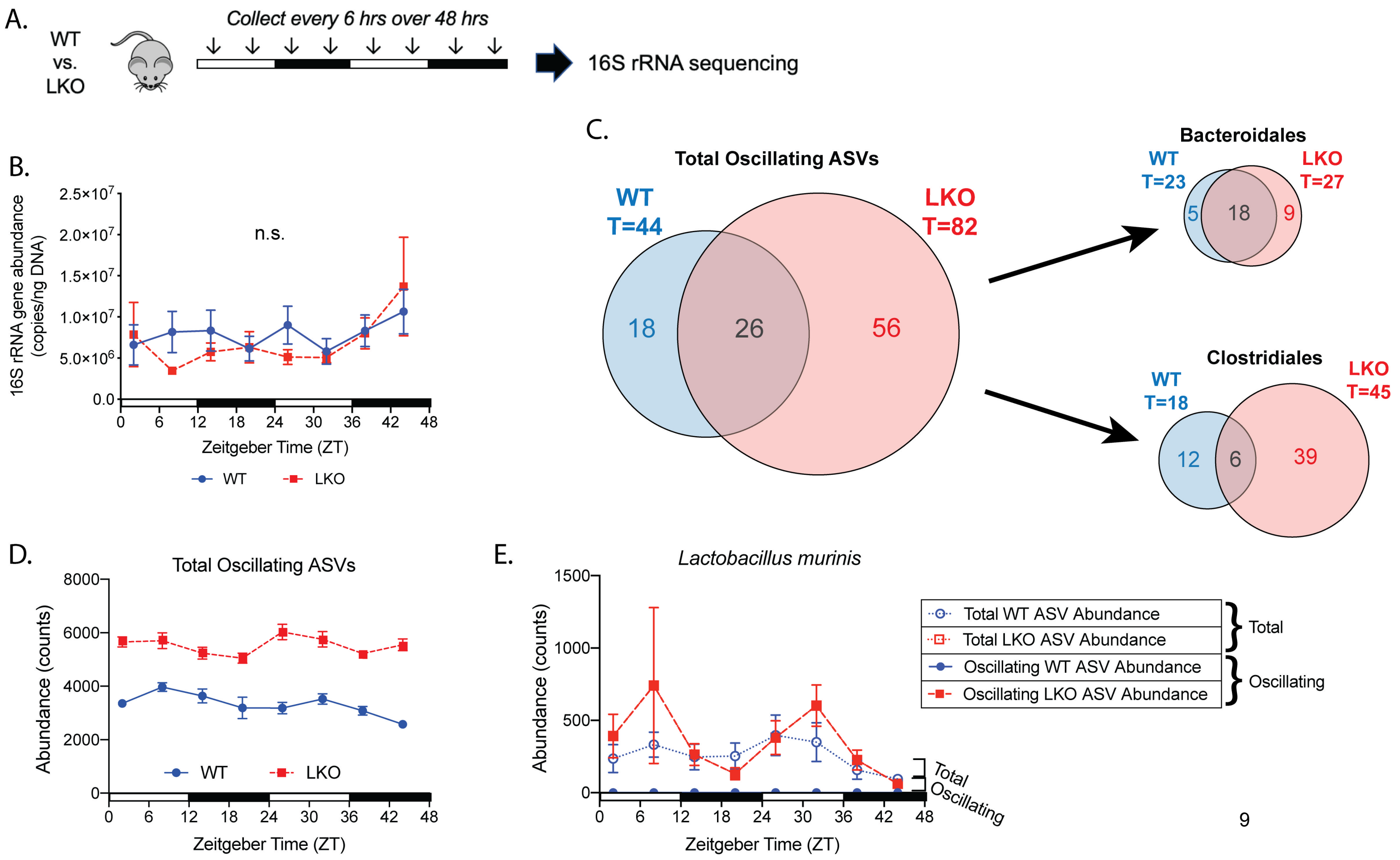

**Supplemental Figure 5, related to Figure 3. Loss of a functional liver clock in female mice results in no change in oscillating ASVs.**

16S rRNA gene sequencing analysis of stool sampled from WT and LKO female mice every 6 hrs over two consecutive 24-hr LD cycles (8 samples per animal) via repeat collections from the same mouse (*n*=8/group). **(A)** 16S rRNA gene abundance measured via qPCR normalized to DNA concentration. **(B)** Proportion of non-oscillating (grey area) vs significantly oscillating (colored areas) Amplicon Sequence Variants (ASVs) identified via eJTK (GammaBH $< 0.05$); oscillating ASVs divided by taxonomic class. **(C)** Venn diagrams presenting total, shared, and unique oscillating ASVs among all taxonomic groups, as well as within specific classes Bacteroidales and Clostridiales. Data points represent mean±SEM. ns=not significant.

## Supplemental Figure 5

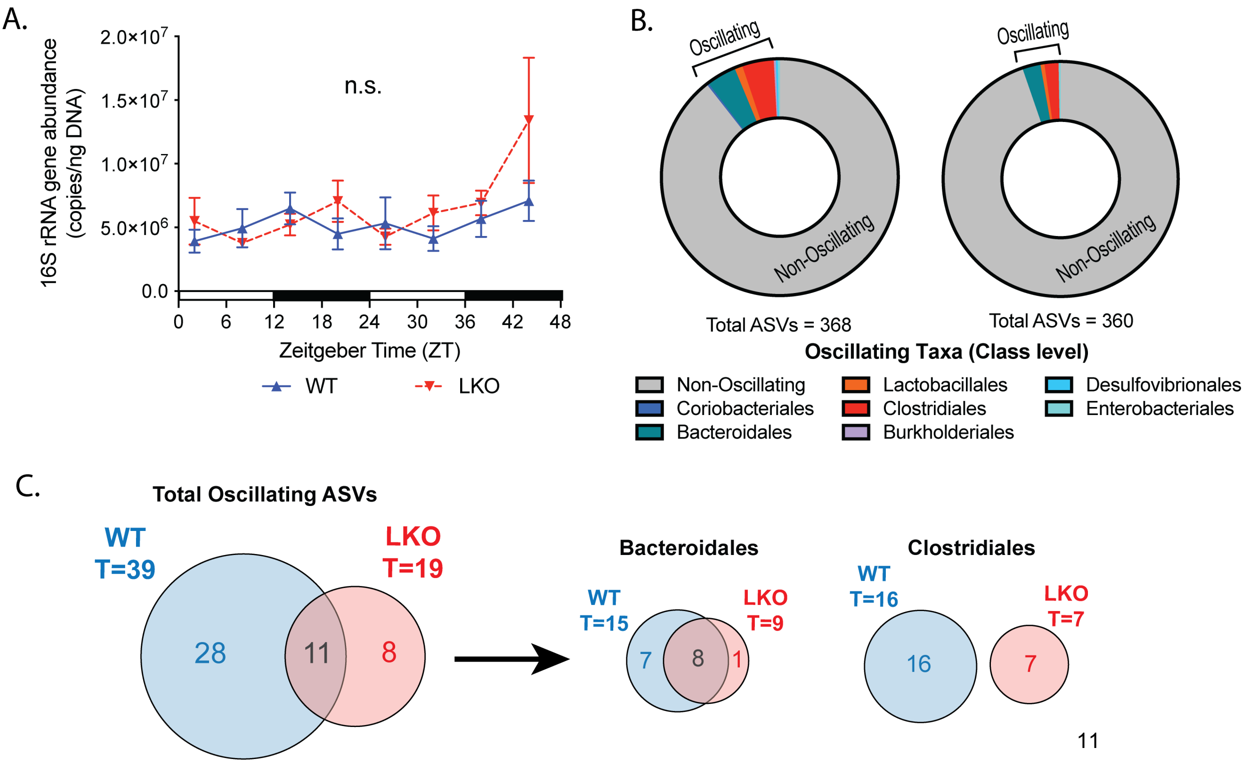

**Supplemental Figure 6, related to Figure 3. Loss of a functional liver clock in male mice results in an increase of oscillating ASVs by log-ratio analysis.**

16S rRNA gene sequencing analysis of stool sampled from WT and LKO male mice every 6 hrs over two consecutive 24-hr LD cycles (8 samples per animal) via repeat collections from the same mouse ($n$=7-8/group). **(A)** Log ratios (calculated by Songbird for each timepoint relative to ZT2) represented as a heatmap with hierarchically sorted ASVs within each taxonomic family. **(B)** Log ratios over time represented with LOESS fitting to visualize trends in each family. Taxa with large numbers of ASVs (Lachnospiraceae and Ruminococcaceae) separated by 3 leaf separations in the hierarchical clustering.

# Supplemental Figure 6

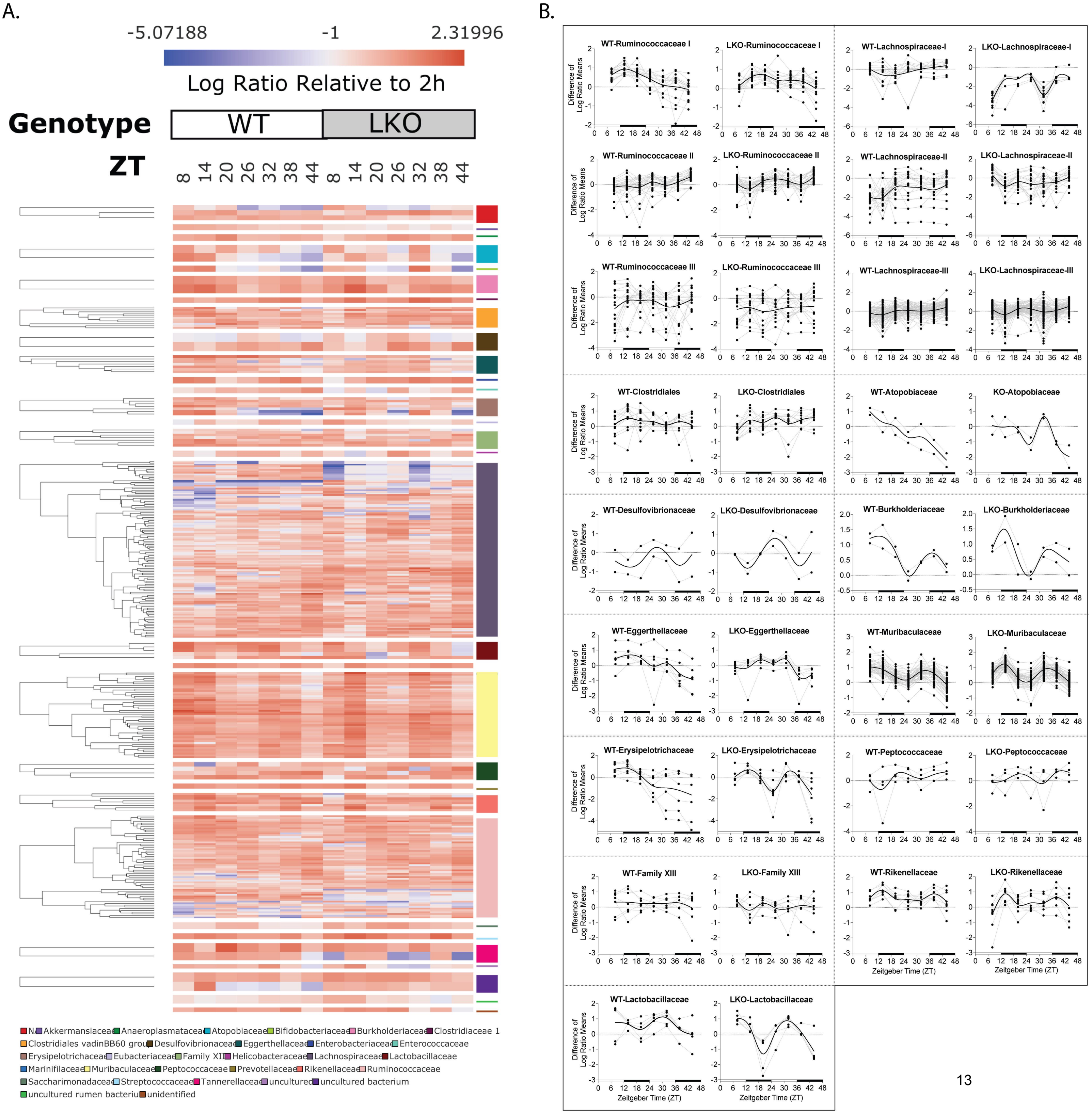

**Supplemental Figure 7, related to Figures 4 and 5. Liver-clock downregulation of metabolic pathways is evident across all timepoints.**

Differential transcriptome analysis of liver samples collected every 4 hrs over 24-hrs from animals maintained in 12:12 LD (ZT2, 6, 10, 14, 18, 22) from SPF and GF, WT and LKO male mice (n=3/timepoint/group). **(A)** Principal Component Analysis of transcriptome profiles, samples divided by collection timepoint. **(B)** Differentially regulated KEGG pathways between WT and LKO, within SPF and GF, divided by ZT collection timepoint. Metabolic pathways are highlighted according to the legend, and non-metabolic pathways are colored gray. **(C)** WT-median-normalized expression of differentially expressed (DE) genes within identified KEGG pathways.

## Supplemental Figure 7

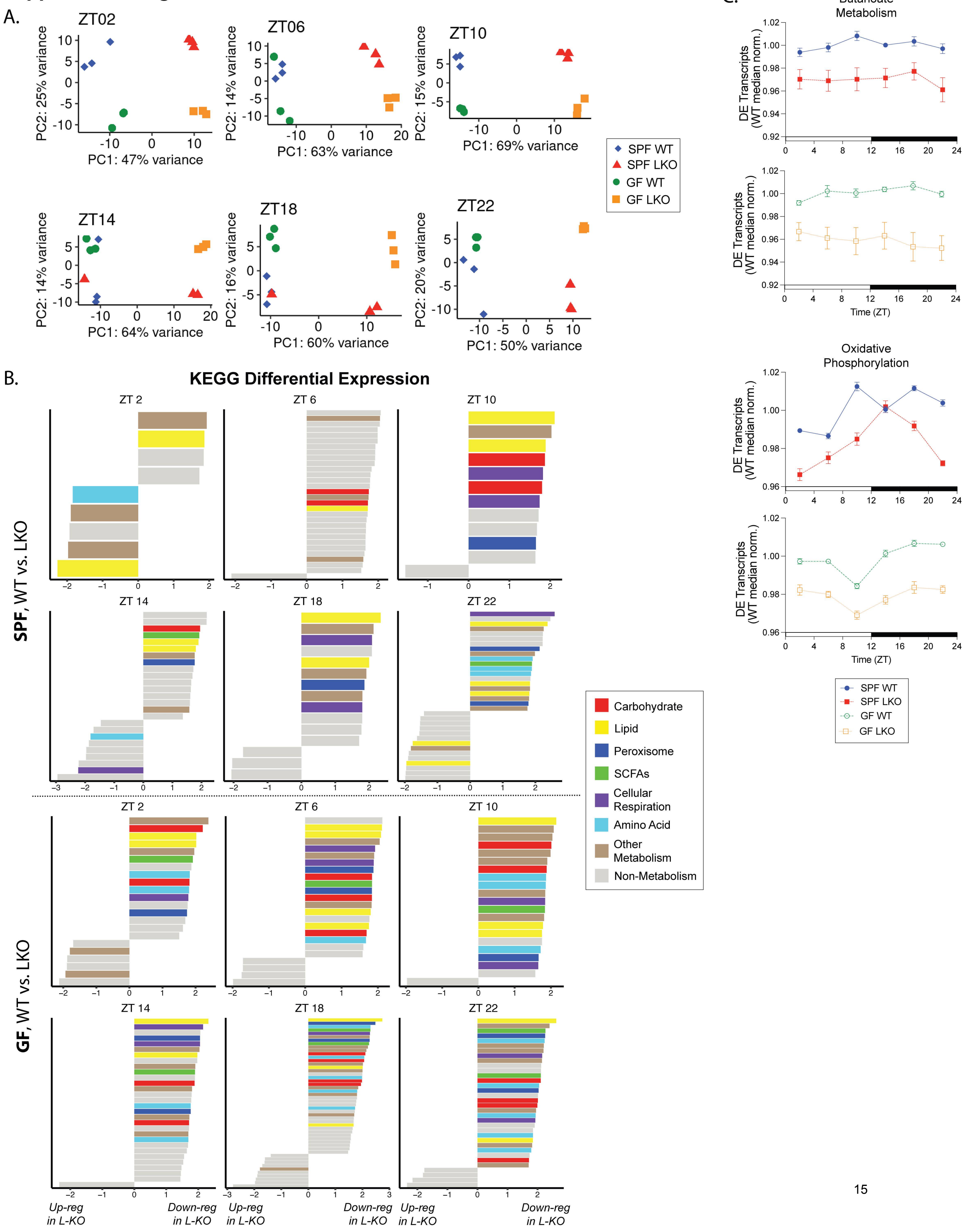

**Supplemental Figure 8, related to Figures 6, 7, and 8. Liver clock drives increases in unique hepatic transcript oscillations and co-occurrence expression in the presence of gut microbes.**

Diurnal and network transcriptome analysis of liver samples collected every 4 hrs over 24-hrs from animals maintained in 12:12 LD (ZT2, 6, 10, 14, 18, 22) from SPF and GF, WT and LKO male mice (n=3/timepoint/group). **(A)** Transcript expression profiles of significantly oscillating SPF WT transcripts; expression values normalized by median, transcripts ordered by time of maximum expression and phase. **(B-E)** Network co-occurrence analysis of correlating transcripts over time within each group ($p<0.001$). **(B)** Network co-occurrence statistics. **(C-E)** Venn diagrams summarizing correlating transcripts (nodes) and connections (edges) that annotate to carbohydrate **(C)**, lipid **(D)**, and amino acid **(E)** metabolic pathways by KEGG, and crossover within SPF and GF conditions; venn diagram sizes are proportional within each sub-figure.

## Supplemental Figure 8

A. **Hepatic Expression Levels of Oscillating SPF WT Transcripts**

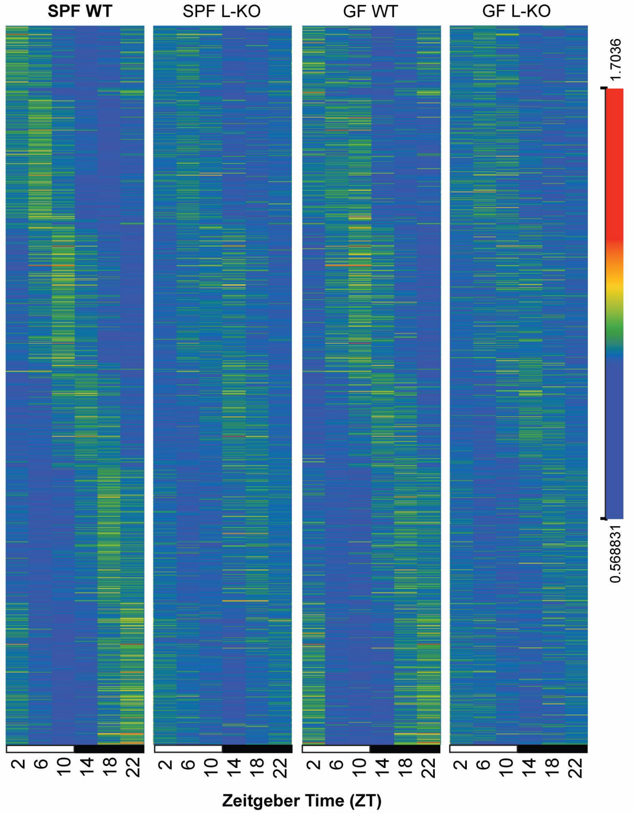


B.

| | SPF WT | SPF LKO | GF WT | GF LKO |
|---|---|---|---|---|
| Number of nodes | 5603 | 6116 | 4623 | 4482 |
| Number of edges | 20922 | 36702 | 10638 | 13464 |
| Avg. number of neighbors | 8.811 | 13.836 | 5.665 | 7.207 |
| Network diameter | 36 | 33 | 30 | 27 |
| Network radius | 18 | 17 | 17 | 17 |
| Characteristic path length | 7.072 | 6.587 | 9.787 | 9.073 |
| Clustering coefficient | 0.225 | 0.25 | 0.218 | 0.248 |
| Network density | 0.002 | 0.003 | 0.002 | 0.002 |
| Network heterogeneity | 1.3 | 1.566 | 1.101 | 1.179 |
| Network centralization | 0.021 | 0.036 | 0.013 | 0.015 |
| Connected components | 410 | 348 | 462 | 354 |

C.

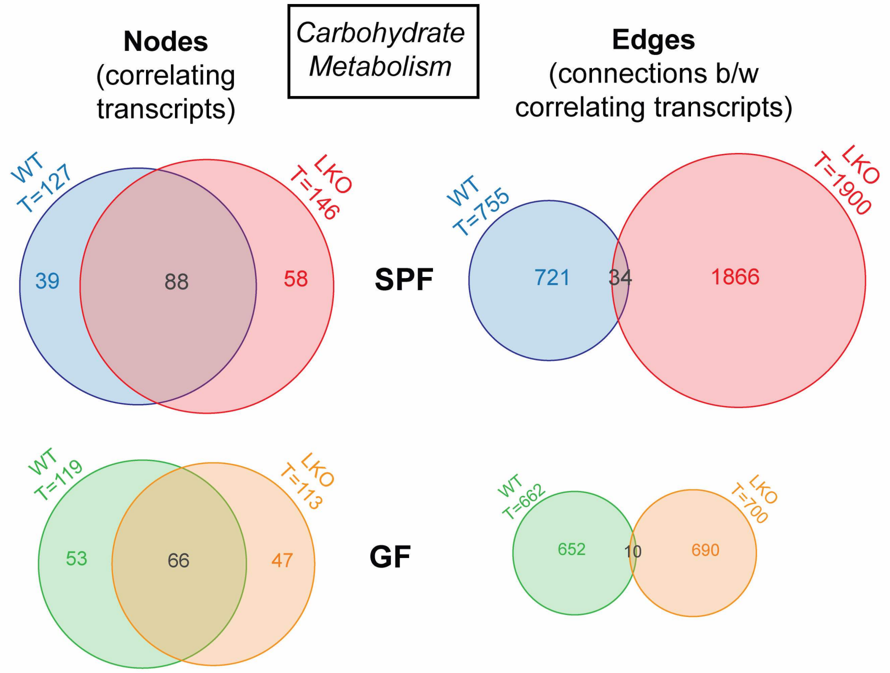


D.

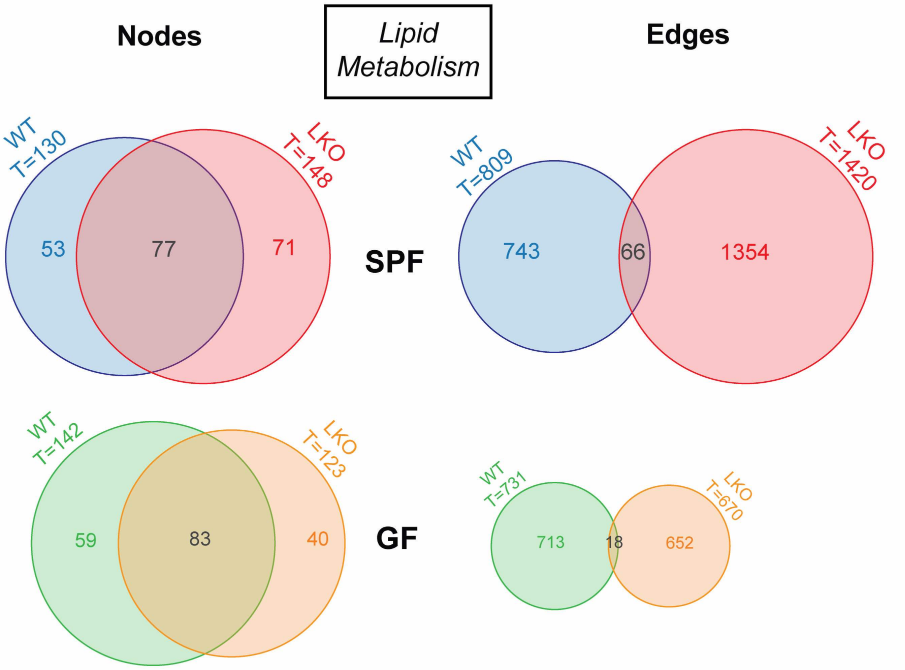


E.

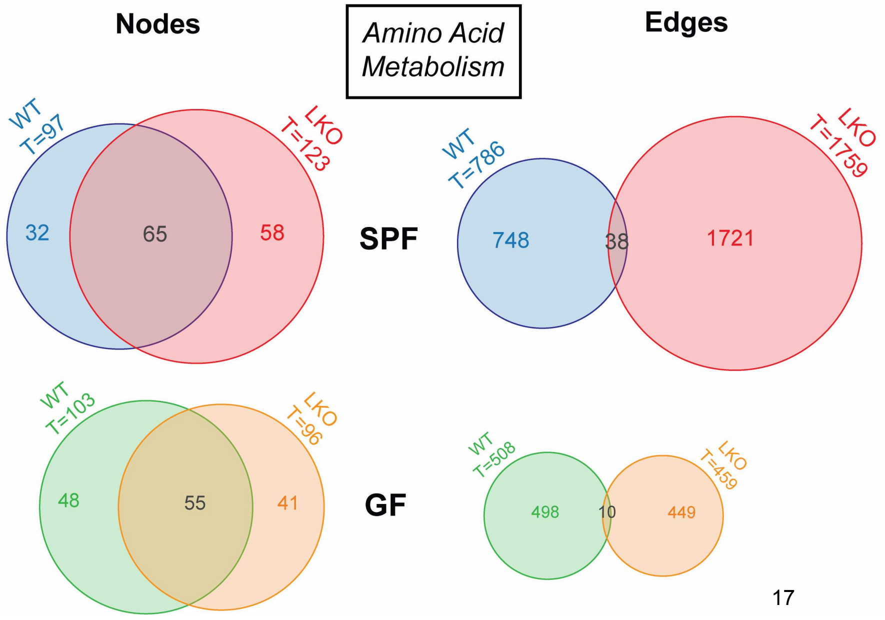

**Supplemental Figure 9, related to Figure 9. Liver clock and gut microbes convey modest changes in food intake patterns and locomotor activity.**

Indirect calorimetry assessment of SPF and GF, WT and LKO male mice, measured over 4 consecutive 12:12 LD cycles (n=12-13). **(A)** Basal metabolic rate represented as kcal/hr. **(B)** Total grams of food intake per LD cycle. **(C)** Food intake summarized as grams consumed per hour; arrows indicate time of feeding onset during the active period. **(D)** Ambulatory motion measured by meters. **(E)** Total ambulatory motion measured by meters per LD cycle. Data points and bars represent mean±SEM, box plots represent median±min/max. Two-tailed unpaired Welch's t tests were performed between 2 groups; *$p<0.05$, ns=not significant.

# Supplemental Figure 9

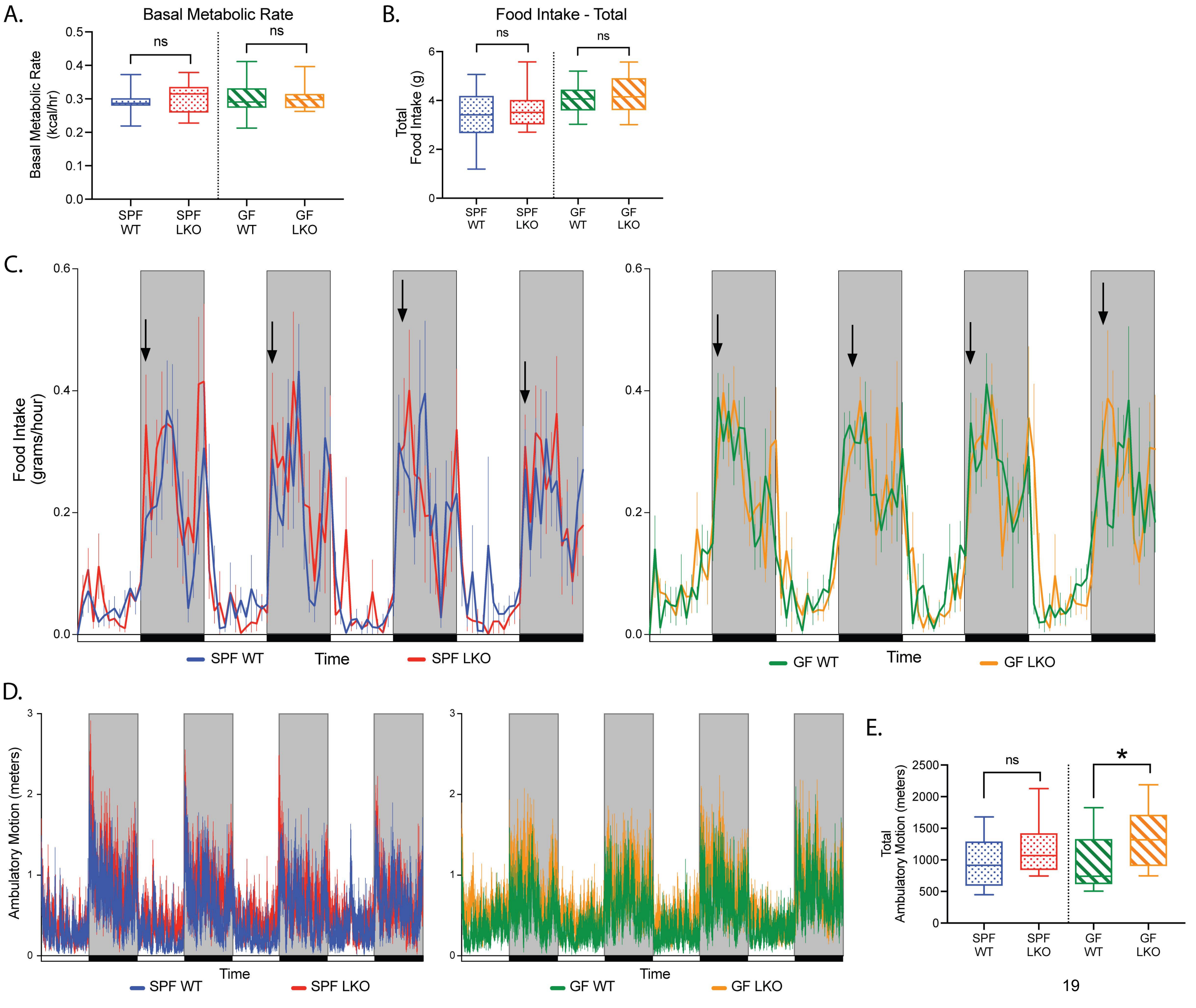

**Supplemental Table 1:** CORUM, GO, KEGG, Reactome, and Wiki pathway enrichment of oscillating hepatic transcripts within SPF and GF, WT and LKO male mice (q-value<0.05 indicated by **X**, analysis via Metascape). **Related to Figure 7**.

| Category | Subcategory | Pathway | SPF | | GF | |
|---|---|---|---|---|---|---|
| | | | WT | L-KO | WT | L-KO |
| Circadian | | CORUM_CLOCK-BMAL1-CRY1_COMPLEX | X | | X | |
| | | CORUM_CLOCK-BMAL1-CRY2_COMPLEX | X | | X | |
| | | GO_CIRCADIAN_REGULATION_OF_GENE_EXPRESSION | X | X | X | X |
| | | GO_CIRCADIAN_RHYTHM | X | | X | |
| | | GO_REGULATION_OF_CIRCADIAN_RHYTHM | X | | X | |
| | | GO_RHYTHMIC_PROCESS | X | | X | |
| | | KEGG_CIRCADIAN_RHYTHM | X | | X | |
| Carbohydrate Metabolism | Insulin | GO_CELLULAR_RESPONSE_TO_INSULIN_STIMULUS | | X | X | X |
| | | GO_INSULIN_RECEPTOR_SIGNALING_PATHWAY | | X | | X |
| | | GO_INSULIN_SECRETION | | | X | |
| | | GO_NEGATIVE_REGULATION_OF_CELLULAR_RESPONSE_TO_INSULIN_STIMULUS | | | | X |
| | | GO_NEGATIVE_REGULATION_OF_INSULIN_RECEPTOR_SIGNALING_PATHWAY | | | | X |
| | | GO_REGULATION_OF_CELLULAR_RESPONSE_TO_INSULIN_STIMULUS | | | | X |
| | | GO_REGULATION_OF_INSULIN_RECEPTOR_SIGNALING_PATHWAY | | X | | X |
| | | GO_REGULATION_OF_INSULIN_SECRETION | | | X | |
| | | GO_RESPONSE_TO_INSULIN | | X | X | X |
| | Glucose/Glycogen/Pyruvate | GO_CELLULAR_GLUCOSE_HOMEOSTASIS | | | X | X |
| | | GO_CELLULAR_RESPONSE_TO_GLUCOSE_STIMULUS | | | X | |
| | | GO_GLUCOSE_HOMEOSTASIS | | X | X | X |
| | | GO_GLUCOSE_IMPORT | | | X | |
| | | GO_GLUCOSE_METABOLIC_PROCESS | X | | X | X |
| | | GO_GLYCOGEN_BIOSYNTHETIC_PROCESS | X | | | X |
| | | GO_GLYCOGEN_METABOLIC_PROCESS | X | | X | X |
| | | GO_REGULATION_OF_GLUCOSE_METABOLIC_PROCESS | X | | X | X |
| | | GO_RESPONSE_TO_GLUCOSE | | | X | X |
| | | KEGG_2-OXOCARBOXYLIC_ACID_METABOLISM | X | | X | |
| | | REACTOME_PYRUVATE_METABOLISM | X | | | |
| | | REACTOME_PYRUVATE_METABOLISM_AND_CITRIC_ACID_(TCA)_CYCLE | X | | X | |
| | 5-carbon Sugars | GO_DEOXYRIBOSE_PHOSPHATE_CATABOLIC_PROCESS | | | X | |
| | | GO_DEOXYRIBOSE_PHOSPHATE_METABOLIC_PROCESS | | | X | |
| | | GO_RIBOSE_PHOSPHATE_BIOSYNTHETIC_PROCESS | | | X | X |
| | | GO_RIBOSE_PHOSPHATE_METABOLIC_PROCESS | | X | X | X |
| | | CORUM_CYTOCHROME_BC1-COMPLEX,_MITOCHONDRIAL | | X | | |
| | | GO_CARBOHYDRATE_DERIVATIVE_BIOSYNTHETIC_PROCESS | X | | X | X |
| | | GO_CARBOHYDRATE_DERIVATIVE_CATABOLIC_PROCESS | | | X | |
| | | GO_CARBOHYDRATE_HOMEOSTASIS | | X | X | X |
| | | GO_CARBOHYDRATE_METABOLIC_PROCESS | X | | X | X |
| | | GO_CARBOHYDRATE_TRANSPORT | X | | X | X |
| | | GO_CELLULAR_CARBOHYDRATE_METABOLIC_PROCESS | X | | X | X |
| | | GO_CELLULAR_GLUCAN_METABOLIC_PROCESS | X | | X | X |
| | | GO_CELLULAR_POLYSACCHARIDE_METABOLIC_PROCESS | | | X | |
| | | GO_CELLULAR_RESPIRATION | | | X | |
| | | GO_CELLULAR_RESPONSE_TO_CARBOHYDRATE_STIMULUS | | | X | X |
| | | GO_CELLULAR_RESPONSE_TO_HEXOSE_STIMULUS | | | X | X |
| | | GO_CELLULAR_RESPONSE_TO_MONOSACCHARIDE_STIMULUS | | | X | X |
| | | GO_CELLULAR_RESPONSE_TO_OXIDATIVE_STRESS | | | X | X |
| | | GO_GANGLIOSIDE_METABOLIC_PROCESS | X | | | |
| | | GO_GLUCAN_BIOSYNTHETIC_PROCESS | X | | | X |
| | | GO_GLUCAN_METABOLIC_PROCESS | X | | X | X |
| | | GO_GLYCOSYL_COMPOUND_BIOSYNTHETIC_PROCESS | | | X | X |
| | | GO_GLYCOSYL_COMPOUND_CATABOLIC_PROCESS | | | X | |
| | | GO_GLYCOSYL_COMPOUND_METABOLIC_PROCESS | X | | X | X |
| | | GO_MONOCARBOXYLIC_ACID_BIOSYNTHETIC_PROCESS | X | | X | X |
| | | GO_MONOSACCHARIDE_BIOSYNTHETIC_PROCESS | | | X | |
| | | GO_MONOSACCHARIDE_METABOLIC_PROCESS | X | X | X | X |
| | | GO_MONOSACCHARIDE_TRANSMEMBRANE_TRANSPORT | | | | X |
| | | GO_REGULATION_OF_CARBOHYDRATE_METABOLIC_PROCESS | | | X | |
| | | GO_REGULATION_OF_CELLULAR_CARBOHYDRATE_METABOLIC_PROCESS | | | X | |
| | | GO_RESPONSE_TO_CARBOHYDRATE | | | X | X |
| | | GO_RESPONSE_TO_MONOSACCHARIDE | | | X | X |
| | | GO_TRICARBOXYLIC_ACID_CYCLE | | | X | |
| | | GO_TRICARBOXYLIC_ACID_METABOLIC_PROCESS | | | X | |
| | | KEGG_CARBON_METABOLISM | X | | X | |
| | | KEGG_GLYOXYLATE_AND_DICARBOXYLATE_METABOLISM | X | | X | |
| | | KEGG_OXIDATIVE_PHOSPHORYLATION | | X | | |
| | | REACTOME_METABOLISM_OF_CARBOHYDRATES | | | X | |
| | | WIKI_ELECTRON_TRANSPORT_CHAIN | | X | | |

| | | | | | | |
|---|---|---|---|---|---|---|
| | | WIKI_GLUTATHIONE_AND_ONE_CARBON_METABOLISM | X | | X | |
| | | WIKI_ONE_CARBON_METABOLISM | X | | X | |
| | | WIKI_ONE_CARBON_METABOLISM_AND_RELATED_PATHWAYS | X | | X | |
| Lipid Metabolism | Fatty Acids | GO_CELLULAR_KETONE_METABOLIC_PROCESS | X | | X | X |
| | | GO_FATTY_ACID_BETA-OXIDATION | X | | X | X |
| | | GO_FATTY_ACID_BIOSYNTHETIC_PROCESS | X | | X | |
| | | GO_FATTY_ACID_CATABOLIC_PROCESS | X | | X | X |
| | | GO_FATTY_ACID_METABOLIC_PROCESS | X | | X | X |
| | | GO_FATTY_ACID_OXIDATION | X | | X | X |
| | | GO_LONG-CHAIN_FATTY_ACID_METABOLIC_PROCESS | | | X | |
| | | GO_METHYL-BRANCHED_FATTY_ACID_METABOLIC_PROCESS | | | X | |
| | | GO_NEGATIVE_REGULATION_OF_FATTY_ACID_METABOLIC_PROCESS | X | | | X |
| | | GO_REGULATION_OF_CELLULAR_KETONE_METABOLIC_PROCESS | X | | X | X |
| | | GO_REGULATION_OF_FATTY_ACID_BETA-OXIDATION | | | X | X |
| | | GO_REGULATION_OF_FATTY_ACID_METABOLIC_PROCESS | X | | X | X |
| | | GO_REGULATION_OF_FATTY_ACID_OXIDATION | X | | X | X |
| | | GO_VERY_LONG-CHAIN_FATTY_ACID_METABOLIC_PROCESS | | | X | |
| | | KEGG_BIOSYNTHESIS_OF_UNSATURATED_FATTY_ACIDS | X | | | |
| | | KEGG_FATTY_ACID_DEGRADATION | | | X | |
| | | REACTOME_FATTY_ACID_METABOLISM | X | | X | |
| | | REACTOME_ALPHA-LINOLEIC_ACID_(ALA)_METABOLISM | X | | | |
| | | REACTOME_ALPHA-LINOLEIC_(OMEGA3)_AND_LINOLEIC_(OMEGA6)_ACID_METABOLISM | X | | | |
| | | REACTOME_LINOLEIC_ACID_(LA)_METABOLISM | X | | | |
| | | WIKI_FATTY_ACID_BIOSYNTHESIS | | | X | |
| | Cholesterol | GO_CHOLESTEROL_BIOSYNTHETIC_PROCESS | | | X | |
| | | GO_CHOLESTEROL_HOMEOSTASIS | X | | X | X |
| | | GO_CHOLESTEROL_METABOLIC_PROCESS | | | X | |
| | | GO_POSITIVE_REGULATION_OF_CHOLESTEROL_METABOLIC_PROCESS | X | | X | |
| | | GO_REGULATION_OF_CHOLESTEROL_METABOLIC_PROCESS | | | X | |
| | | REACTOME_CHOLESTEROL_BIOSYNTHESIS | | X | X | |
| | | REACTOME_CHOLESTEROL_BIOSYNTHESIS_VIA_DESMOSTEROL | | | X | |
| | | REACTOME_CHOLESTEROL_BIOSYNTHESIS_VIA_LATHOSTEROL | | | X | |
| | | WIKI_CHOLESTEROL_BIOSYNTHESIS | | X | X | |
| | Bile Acids | GO_BILE_ACID_BIOSYNTHETIC_PROCESS | | | X | |
| | | GO_BILE_ACID_METABOLIC_PROCESS | X | | X | X |
| | | KEGG_BILE_SECRETION | X | | X | X |
| | | KEGG_PRIMARY_BILE_ACID_BIOSYNTHESIS | | | X | |
| | | REACTOME_BILE_ACID_AND_BILE_SALT_METABOLISM | | | X | |
| | | REACTOME_SYNTHESIS_OF_BILE_ACIDS_AND_BILE_SALTS | | | X | |
| | | REACTOME_SYNTHESIS_OF_BILE_ACIDS_AND_BILE_SALTS_VIA_24-HYDROXYCHOLESTEROL | | | X | |
| | | REACTOME_SYNTHESIS_OF_BILE_ACIDS_AND_BILE_SALTS_VIA_7ALPHA-HYDROXYCHOLESTEROL | | | X | |
| | Peroxisome | GO_ESTABLISHMENT_OF_PROTEIN_LOCALIZATION_TO_PEROXISOME | | | | X |
| | | GO_PEROXISOMAL_TRANSPORT | | | | X |
| | | GO_PEROXISOME_ORGANIZATION | | | X | |
| | | GO_PROTEIN_LOCALIZATION_TO_PEROXISOME | | | | X |
| | | GO_PROTEIN_TARGETING_TO_PEROXISOME | | | | X |
| | | KEGG_PEROXISOME | X | X | X | X |
| | | KEGG_PPAR_SIGNALING_PATHWAY | X | | X | X |
| | | REACTOME_PEROXISOMAL_LIPID_METABOLISM | | | X | X |
| | | REACTOME_PEROXISOMAL_PROTEIN_IMPORT | | | X | X |
| | | WIKI_PPAR_SIGNALING_PATHWAY | X | | X | |
| | | GO_GLYCEROLIPID_METABOLIC_PROCESS | | X | X | X |
| | | GO_ACYLGLYCEROL_BIOSYNTHETIC_PROCESS | | | X | X |
| | | GO_ACYLGLYCEROL_METABOLIC_PROCESS | | | X | X |
| | | GO_CANALICULAR_BILE_ACID_TRANSPORT | | | | X |
| | | GO_CELLULAR_LIPID_CATABOLIC_PROCESS | X | | X | X |
| | | GO_CELLULAR_RESPONSE_TO_STEROID_HORMONE_STIMULUS | | | | X |
| | | GO_CELLULAR_RESPONSE_TO_STEROL | | | X | X |
| | | GO_DITERPENOID_METABOLIC_PROCESS | | | X | |
| | | GO_FATTY-ACYL-COA_METABOLIC_PROCESS | | | X | X |
| | | GO_GLYCEROLIPID_BIOSYNTHETIC_PROCESS | | X | X | X |
| | | GO_GLYCEROPHOSPHOLIPID_BIOSYNTHETIC_PROCESS | | | | X |
| | | GO_GLYCEROPHOSPHOLIPID_METABOLIC_PROCESS | | | | X |
| | | GO_GLYCOSPHINGOLIPID_METABOLIC_PROCESS | X | | | |
| | | GO_ISOPRENOID_METABOLIC_PROCESS | | | X | |
| | | GO_LIPID_BIOSYNTHETIC_PROCESS | X | X | X | X |
| | | GO_LIPID_CATABOLIC_PROCESS | X | | X | X |
| | | GO_LIPID_DROPLET_ORGANIZATION | X | | X | |
| | | GO_LIPID_HOMEOSTASIS | | | X | X |
| | | GO_LIPID_LOCALIZATION | X | | X | X |
| | | GO_LIPID_MODIFICATION | X | | X | X |

| | | | | | |
|---|---|---|---|---|---|
| | GO_LIPID_OXIDATION | X | | X | X |
| | GO_LIPID_PHOSPHORYLATION | | | | X |
| | GO_LIPID_STORAGE | X | | X | |
| | GO_LIPID_TRANSPORT | X | | X | X |
| | GO_LIPOPROTEIN_METABOLIC_PROCESS | | | | X |
| | GO_LONG-CHAIN_FATTY-ACYL-COA_METABOLIC_PROCESS | | | X | X |
| | GO_MEMBRANE_LIPID_METABOLIC_PROCESS | | | X | |
| | GO_NEGATIVE_REGULATION_OF_GLUCOCORTICOID_RECEPTOR_SIGNALING_PATHWAY | X | | X | |
| | GO_NEGATIVE_REGULATION_OF_LIPID_LOCALIZATION | X | | X | |
| | GO_NEUTRAL_LIPID_BIOSYNTHETIC_PROCESS | | | X | X |
| | GO_NEUTRAL_LIPID_CATABOLIC_PROCESS | | | X | |
| | GO_NEUTRAL_LIPID_METABOLIC_PROCESS | | | X | X |
| | GO_PHOSPHATIDIC_ACID_METABOLIC_PROCESS | | | | X |
| | GO_PHOSPHATIDYLCHOLINE_BIOSYNTHETIC_PROCESS | | | X | |
| | GO_PHOSPHOLIPID_BIOSYNTHETIC_PROCESS | | X | X | X |
| | GO_PHOSPHOLIPID_METABOLIC_PROCESS | | X | X | X |
| | GO_POSITIVE_REGULATION_OF_LIPID_BIOSYNTHETIC_PROCESS | X | X | X | X |
| | GO_POSITIVE_REGULATION_OF_LIPID_METABOLIC_PROCESS | X | | X | X |
| | GO_POSITIVE_REGULATION_OF_STEROID_METABOLIC_PROCESS | X | | | |
| | GO_POSITIVE_REGULATION_OF_TRIGLYCERIDE_BIOSYNTHETIC_PROCESS | | | X | X |
| | GO_POSITIVE_REGULATION_OF_TRIGLYCERIDE_METABOLIC_PROCESS | | | X | X |
| | GO_REGULATION_OF_LIPID_BIOSYNTHETIC_PROCESS | X | X | X | X |
| | GO_REGULATION_OF_LIPID_CATABOLIC_PROCESS | | | X | X |
| | GO_REGULATION_OF_LIPID_LOCALIZATION | X | | X | |
| | GO_REGULATION_OF_LIPID_METABOLIC_PROCESS | X | X | X | X |
| | GO_REGULATION_OF_LIPID_TRANSPORT | | | X | |
| | GO_REGULATION_OF_PHOSPHOLIPID_METABOLIC_PROCESS | | | | X |
| | GO_REGULATION_OF_STEROID_METABOLIC_PROCESS | | X | X | |
| | GO_REGULATION_OF_TRIGLYCERIDE_BIOSYNTHETIC_PROCESS | | | X | X |
| | GO_REGULATION_OF_TRIGLYCERIDE_METABOLIC_PROCESS | | | X | X |
| | GO_RESPONSE_TO_STEROID_HORMONE | | | | X |
| | GO_RESPONSE_TO_STEROL | | | X | |
| | GO_SPHINGOLIPID_CATABOLIC_PROCESS | X | | | |
| | GO_SPHINGOLIPID_METABOLIC_PROCESS | X | | X | |
| | GO_STEROID_BIOSYNTHETIC_PROCESS | | X | X | |
| | GO_STEROID_METABOLIC_PROCESS | X | X | X | X |
| | GO_STEROL_BIOSYNTHETIC_PROCESS | | | X | |
| | GO_STEROL_HOMEOSTASIS | X | | X | X |
| | GO_STEROL_METABOLIC_PROCESS | | | X | |
| | GO_TERPENOID_METABOLIC_PROCESS | | | X | |
| | GO_TRIGLYCERIDE_BIOSYNTHETIC_PROCESS | | | X | X |
| | GO_TRIGLYCERIDE_METABOLIC_PROCESS | | | X | X |
| | KEGG_GLYCEROLIPID_METABOLISM | | | X | X |
| | KEGG_GLYCEROPHOSPHOLIPID_METABOLISM | | | X | X |
| | KEGG_PHOSPHATIDYLINOSITOL_SIGNALING_SYSTEM | | | | X |
| | KEGG_STEROID_BIOSYNTHESIS | | | X | |
| | KEGG_TERPENOID_BACKBONE_BIOSYNTHESIS | | | X | |
| | KEGG_TRIACYLGLYCEROL_BIOSYNTHESIS | | | X | X |
| | REACTOME_FATTY_ACYL-COA_BIOSYNTHESIS | | | X | |
| | REACTOME_GLYCEROPHOSPHOLIPID_BIOSYNTHESIS | | | X | X |
| | REACTOME_METABOLISM_OF_LIPIDS | X | X | X | X |
| | REACTOME_METABOLISM_OF_STEROIDS | | | X | |
| | REACTOME_PHOSPHOLIPID_METABOLISM | | | X | X |
| | REACTOME_SYNTHESIS_OF_VERY_LONG-CHAIN_FATTY_ACYL-COAS | X | | X | |
| Proteins | GO_CELLULAR_PROTEIN_CATABOLIC_PROCESS | X | X | X | X |
| | GO_GLYCOPROTEIN_BIOSYNTHETIC_PROCESS | | | | X |
| | GO_GLYCOPROTEIN_METABOLIC_PROCESS | | | | X |
| | GO_LIPOPROTEIN_METABOLIC_PROCESS | | | | X |
| | GO_MODIFICATION-DEPENDENT_PROTEIN_CATABOLIC_PROCESS | | X | X | X |
| | GO_POSITIVE_REGULATION_OF_CELLULAR_PROTEIN_CATABOLIC_PROCESS | | | | X |
| | GO_POSITIVE_REGULATION_OF_PROTEIN_CATABOLIC_PROCESS | | | | X |
| | GO_PROTEASOMAL_PROTEIN_CATABOLIC_PROCESS | | X | X | X |
| | GO_PROTEASOME-MEDIATED_UBIQUITIN-DEPENDENT_PROTEIN_CATABOLIC_PROCESS | | X | X | X |
| | GO_PROTEOLYSIS_INVOLVED_IN_CELLULAR_PROTEIN_CATABOLIC_PROCESS | X | X | X | X |
| | GO_REGULATION_OF_CELLULAR_PROTEIN_CATABOLIC_PROCESS | | | | X |
| | GO_REGULATION_OF_LIPOPROTEIN_PARTICLE_CLEARANCE | | | X | |
| | GO_REGULATION_OF_PROTEASOMAL_PROTEIN_CATABOLIC_PROCESS | | | | X |
| | GO_REGULATION_OF_PROTEASOMAL_UBIQUITIN-DEPENDENT_PROTEIN_CATABOLIC_PROCESS | | | | X |
| | GO_REGULATION_OF_PROTEIN_CATABOLIC_PROCESS | | | X | X |
| | GO_REGULATION_OF_PROTEOLYSIS_INVOLVED_IN_CELLULAR_PROTEIN_CATABOLIC_PROCESS | | | | X |
| | GO_SCF-DEPENDENT_PROTEASOMAL_UBIQUITIN-DEPENDENT_PROTEIN_CATABOLIC_PROCESS | | | | X |

| | | | | | | |
|---|---|---|---|---|---|---|
| Protein Metabolism | | GO_UBIQUITIN-DEPENDENT_PROTEIN_CATABOLIC_PROCESS | | | X | X |
| | Peptides AA | GO_ALPHA-AMINO_ACID_BIOSYNTHETIC_PROCESS | | | X | |
| | | GO_ALPHA-AMINO_ACID_CATABOLIC_PROCESS | X | | X | |
| | | GO_ALPHA-AMINO_ACID_METABOLIC_PROCESS | X | X | X | X |
| | | GO_AMINO_ACID_ACTIVATION | | | | X |
| | | GO_AROMATIC_AMINO_ACID_FAMILY_CATABOLIC_PROCESS | X | | X | |
| | | GO_ASPARTATE_FAMILY_AMINO_ACID_BIOSYNTHETIC_PROCESS | | | X | |
| | | GO_ASPARTATE_FAMILY_AMINO_ACID_CATABOLIC_PROCESS | | | X | |
| | | GO_ASPARTATE_FAMILY_AMINO_ACID_METABOLIC_PROCESS | X | | X | |
| | | GO_CELLULAR_AMINO_ACID_BIOSYNTHETIC_PROCESS | | | X | |
| | | GO_CELLULAR_AMINO_ACID_CATABOLIC_PROCESS | X | | X | |
| | | GO_CELLULAR_AMINO_ACID_METABOLIC_PROCESS | X | X | X | X |
| | | GO_CELLULAR_MODIFIED_AMINO_ACID_METABOLIC_PROCESS | X | | X | |
| | | GO_CELLULAR_RESPONSE_TO_AMINO_ACID_STARVATION | | | | X |
| | | GO_CELLULAR_RESPONSE_TO_PEPTIDE | | X | X | X |
| | | GO_CELLULAR_RESPONSE_TO_PEPTIDE_HORMONE_STIMULUS | | X | X | X |
| | | GO_CYSTEINE_CATABOLIC_PROCESS | | | X | |
| | | GO_CYSTEINE_METABOLIC_PROCESS | | | X | |
| | | GO_GLUTAMINE_FAMILY_AMINO_ACID_METABOLIC_PROCESS | X | | | |
| | | GO_GLUTAMINE_METABOLIC_PROCESS | | | | X |
| | | GO_GLYCINE_METABOLIC_PROCESS | | | X | |
| | | GO_L-CYSTEINE_CATABOLIC_PROCESS | | | X | |
| | | GO_L-CYSTEINE_METABOLIC_PROCESS | | | X | |
| | | GO_METHIONINE_BIOSYNTHETIC_PROCESS | X | | | |
| | | GO_METHIONINE_METABOLIC_PROCESS | X | | X | |
| | | GO_NEGATIVE_REGULATION_OF_PROTEIN_SERINE/THREONINE_KINASE_ACTIVITY | | | | X |
| | | GO_ORNITHINE_METABOLIC_PROCESS | | X | | |
| | | GO_PEPTIDE_BIOSYNTHETIC_PROCESS | | X | X | X |
| | | GO_PEPTIDE_HORMONE_SECRETION | | | X | |
| | | GO_PEPTIDE_SECRETION | | | X | |
| | | GO_PEPTIDYL-ASPARAGINE_MODIFICATION | | | | X |
| | | GO_PROTEIN_N-LINKED_GLYCOSYLATION_VIA_ASPARAGINE | | | | X |
| | | GO_REGULATION_OF_CYCLIN-DEPENDENT_PROTEIN_SERINE/THREONINE_KINASE_ACTIVITY | | | X | |
| | | GO_REGULATION_OF_PEPTIDE_HORMONE_SECRETION | | | X | |
| | | GO_REGULATION_OF_PEPTIDE_TRANSPORT | | X | X | X |
| | | GO_RESPONSE_TO_AMINO_ACID_STARVATION | | | X | X |
| | | GO_RESPONSE_TO_PEPTIDE | | X | X | X |
| | | GO_RESPONSE_TO_PEPTIDE_HORMONE | | X | X | X |
| | | GO_S-ADENOSYLHOMOCYSTEINE_METABOLIC_PROCESS | | | X | |
| | | GO_S-ADENOSYLMETHIONINE_METABOLIC_PROCESS | | | X | |
| | | GO_SERINE_FAMILY_AMINO_ACID_CATABOLIC_PROCESS | | | X | |
| | | GO_SERINE_FAMILY_AMINO_ACID_METABOLIC_PROCESS | X | | X | |
| | | GO_SULFUR_AMINO_ACID_BIOSYNTHETIC_PROCESS | X | | | |
| | | GO_SULFUR_AMINO_ACID_CATABOLIC_PROCESS | | | X | |
| | | GO_SULFUR_AMINO_ACID_METABOLIC_PROCESS | X | | X | |
| | | GO_TAURINE_METABOLIC_PROCESS | | | X | |
| | | GO_TRANSMEMBRANE_RECEPTOR_PROTEIN_TYROSINE_KINASE_SIGNALING_PATHWAY | | | | X |
| | | KEGG_ARGININE_BIOSYNTHESIS | X | | X | |
| | | KEGG_BIOSYNTHESIS_OF_AMINO_ACIDS | X | | X | |
| | | KEGG_CYSTEINE_AND_METHIONINE_METABOLISM | X | | X | |
| | | KEGG_GLYCINE,_SERINE_AND_THREONINE_METABOLISM | | | X | |
| | | KEGG_LYSINE_DEGRADATION | | | X | |
| | | KEGG_LYSINE_DEGRADATION,_LYSINE_=>_SACCHAROPINE_=>_ACETOACETYL-COA | | | X | |
| | | KEGG_METHIONINE_DEGRADATION | | | X | |
| | | KEGG_TRYPTOPHAN_METABOLISM | | | X | |
| | | REACTOME_ASPARAGINE_N-LINKED_GLYCOSYLATION | X | X | X | X |
| | | REACTOME_GLYOXYLATE_METABOLISM_AND_GLYCINE_DEGRADATION | X | | | |
| | | REACTOME_METABOLISM_OF_AMINO_ACIDS_AND_DERIVATIVES | X | X | X | X |
| | | REACTOME_OXYGEN-DEPENDENT_PROLINE_HYDROXYLATION_OF_HYPOXIA-INDUCIBLE_FACTOR_ALPHA | | | X | |
| | | REACTOME_SULFUR_AMINO_ACID_METABOLISM | | | X | X |
| | | WIKI_TRYPTOPHAN_METABOLISM | | | X | |
| | | GO_2'-DEOXYRIBONUCLEOTIDE_METABOLIC_PROCESS | | | X | |
| | | GO_DEOXYRIBONUCLEOSIDE_MONOPHOSPHATE_METABOLIC_PROCESS | X | | | |
| | | GO_DEOXYRIBONUCLEOTIDE_METABOLIC_PROCESS | | | X | |
| | | GO_DNA_BIOSYNTHETIC_PROCESS | | X | | |
| | | GO_MRNA_CATABOLIC_PROCESS | | | | X |
| | | GO_MRNA_METABOLIC_PROCESS | | | | X |
| | | GO_NCRNA_METABOLIC_PROCESS | | | X | X |

| | | | | | |
|---|---|---|---|---|---|
| Nucleotide Metabolism | GO_NICOTINAMIDE_NUCLEOTIDE_BIOSYNTHETIC_PROCESS | | | | X |
| | GO_NICOTINAMIDE_NUCLEOTIDE_METABOLIC_PROCESS | | | X | X |
| | GO_NUCLEAR-TRANSCRIBED_MRNA_CATABOLIC_PROCESS | | | | X |
| | GO_NUCLEOBASE-CONTAINING_COMPOUND_CATABOLIC_PROCESS | | | X | X |
| | GO_NUCLEOBASE-CONTAINING_SMALL_MOLECULE_BIOSYNTHETIC_PROCESS | | | X | X |
| | GO_NUCLEOBASE-CONTAINING_SMALL_MOLECULE_METABOLIC_PROCESS | X | X | X | X |
| | GO_NUCLEOSIDE_BIOSYNTHETIC_PROCESS | | | X | |
| | GO_NUCLEOSIDE_BISPHOSPHATE_BIOSYNTHETIC_PROCESS | | | X | X |
| | GO_NUCLEOSIDE_BISPHOSPHATE_METABOLIC_PROCESS | X | X | X | X |
| | GO_NUCLEOSIDE_METABOLIC_PROCESS | X | | X | X |
| | GO_NUCLEOSIDE_MONOPHOSPHATE_METABOLIC_PROCESS | | X | X | |
| | GO_NUCLEOSIDE_PHOSPHATE_BIOSYNTHETIC_PROCESS | X | | X | X |
| | GO_NUCLEOSIDE_PHOSPHATE_METABOLIC_PROCESS | X | X | X | X |
| | GO_NUCLEOSIDE_TRIPHOSPHATE_METABOLIC_PROCESS | | X | X | |
| | GO_NUCLEOTIDE_BIOSYNTHETIC_PROCESS | X | | X | X |
| | GO_NUCLEOTIDE_METABOLIC_PROCESS | X | X | X | X |
| | GO_POSITIVE_REGULATION_OF_MRNA_METABOLIC_PROCESS | | | | X |
| | GO_PTERIDINE-CONTAINING_COMPOUND_METABOLIC_PROCESS | | | X | |
| | GO_PURINE_DEOXYRIBONUCLEOSIDE_TRIPHOSPHATE_METABOLIC_PROCESS | | | X | |
| | GO_PURINE_DEOXYRIBONUCLEOTIDE_METABOLIC_PROCESS | | | X | |
| | GO_PURINE_NUCLEOSIDE_BISPHOSPHATE_BIOSYNTHETIC_PROCESS | | | X | X |
| | GO_PURINE_NUCLEOSIDE_BISPHOSPHATE_METABOLIC_PROCESS | X | X | X | X |
| | GO_PURINE_NUCLEOSIDE_CATABOLIC_PROCESS | | | X | |
| | GO_PURINE_NUCLEOSIDE_METABOLIC_PROCESS | | | X | X |
| | GO_PURINE_NUCLEOSIDE_MONOPHOSPHATE_METABOLIC_PROCESS | | X | X | |
| | GO_PURINE_NUCLEOSIDE_TRIPHOSPHATE_METABOLIC_PROCESS | | X | X | |
| | GO_PURINE_NUCLEOTIDE_BIOSYNTHETIC_PROCESS | | | X | X |
| | GO_PURINE_NUCLEOTIDE_METABOLIC_PROCESS | | X | X | X |
| | GO_PURINE_RIBONUCLEOSIDE_CATABOLIC_PROCESS | | | X | |
| | GO_PURINE_RIBONUCLEOSIDE_METABOLIC_PROCESS | X | | X | X |
| | GO_PURINE_RIBONUCLEOSIDE_MONOPHOSPHATE_METABOLIC_PROCESS | | X | X | |
| | GO_PURINE_RIBONUCLEOSIDE_TRIPHOSPHATE_METABOLIC_PROCESS | | X | X | |
| | GO_PURINE_RIBONUCLEOTIDE_BIOSYNTHETIC_PROCESS | | | X | X |
| | GO_PURINE_RIBONUCLEOTIDE_METABOLIC_PROCESS | | X | X | X |
| | GO_PURINE-CONTAINING_COMPOUND_BIOSYNTHETIC_PROCESS | | | X | |
| | GO_PURINE-CONTAINING_COMPOUND_CATABOLIC_PROCESS | | | X | |
| | GO_PURINE-CONTAINING_COMPOUND_METABOLIC_PROCESS | X | X | X | X |
| | GO_PYRIDINE_NUCLEOTIDE_METABOLIC_PROCESS | | | X | |
| | GO_PYRIDINE-CONTAINING_COMPOUND_BIOSYNTHETIC_PROCESS | | | | X |
| | GO_PYRIDINE-CONTAINING_COMPOUND_METABOLIC_PROCESS | X | | X | |
| | GO_PYRIMIDINE_NUCLEOSIDE_METABOLIC_PROCESS | | X | | |
| | GO_PYRIMIDINE-CONTAINING_COMPOUND_BIOSYNTHETIC_PROCESS | | | X | |
| | GO_PYRIMIDINE-CONTAINING_COMPOUND_METABOLIC_PROCESS | | X | | |
| | GO_REGULATION_OF_DNA_METABOLIC_PROCESS | | | | X |
| | GO_REGULATION_OF_MRNA_CATABOLIC_PROCESS | | | | X |
| | GO_REGULATION_OF_MRNA_METABOLIC_PROCESS | | | | X |
| | GO_RIBONUCLEOSIDE_BIOSYNTHETIC_PROCESS | | | X | |
| | GO_RIBONUCLEOSIDE_BISPHOSPHATE_BIOSYNTHETIC_PROCESS | | | X | X |
| | GO_RIBONUCLEOSIDE_BISPHOSPHATE_METABOLIC_PROCESS | X | X | X | X |
| | GO_RIBONUCLEOSIDE_METABOLIC_PROCESS | X | | X | X |
| | GO_RIBONUCLEOSIDE_MONOPHOSPHATE_METABOLIC_PROCESS | | X | X | |
| | GO_RIBONUCLEOSIDE_TRIPHOSPHATE_METABOLIC_PROCESS | | X | X | |
| | GO_RIBONUCLEOTIDE_BIOSYNTHETIC_PROCESS | | | X | X |
| | GO_RIBONUCLEOTIDE_METABOLIC_PROCESS | | X | X | X |
| | GO_RNA_CATABOLIC_PROCESS | | | | X |
| | GO_RNA-DEPENDENT_DNA_BIOSYNTHETIC_PROCESS | | X | | |
| | GO_RRNA_METABOLIC_PROCESS | | | X | X |
| | GO_TRNA_METABOLIC_PROCESS | | | | X |
| | KEGG_PURINE_METABOLISM | | | X | X |
| | KEGG_PYRIMIDINE_METABOLISM | X | | | X |
| | REACTOME_METABOLISM_OF_NUCLEOTIDES | X | | X | |
| | REACTOME_METABOLISM_OF_RNA | | | X | X |
| | REACTOME_PURINE_CATABOLISM | | | X | |
| | REACTOME_PYRIMIDINE_SALVAGE | X | | | |
| | WIKI_PURINE_METABOLISM | | | X | X |

**Supplemental Table 2: Key Resources Table,** related to Methods**.**

| REAGENT or RESOURCE | SOURCE | IDENTIFIER |
|---|---|---|
| Antibodies | | |
| Rb pAb to BMAL1 | Abcam | Cat#ab93806<br>RRID#AB_10675117<br>Lot#GR3368431-1 |
| Anti-GAPDH, MAb | Invitrogen | Cat#AM4300<br>RRID#AB_437392<br>Lot#00959883 |
| Anti-mouse IgG, HRP-linked Antibody | Cell Signalling Technology | Cat#7076S<br>RRID#AB_330924<br>Lot#33 |
| Anti-rabbit IgG, HRP-linked Antibody | Cell Signalling Technology | Cat#7074S<br>RRID#AB_2099233<br>Lot#29 |
| Bacterial and Virus Strains | | |
| n/a | | |
| Biological Samples | | |
| n/a | | |
| Chemicals, Peptides, and Recombinant Proteins | | |
| 50% Dextrose Injection, USP | Hospira | Cat#00409-4902-34 |
| Sodium Pyruvate | Sigma-Aldrich | Cat#P5280 |
| Humulin R Insulin | Eli Lilly | Cat#0002-8215-01 |
| pCPT-cAMP | Sigma-Aldrich | Cat#C3912 |
| DMEM low glucose, primary hepatocyte digestion & culture medium | Corning | Cat#10-014-CV |
| DMEM high glucose, primary hepatocyte isolation medium | Gibco | Cat#11960-044 |
| Collagenase Type IV | Worthington Biochemical | Cat#LS004188 |
| PBS with $MgCl_2$ and $CaCl_2$ | Sigma-Aldrich | Cat#D8662 |
| HEPES buffer | Thermo Scientific | Cat#15630080 |
| DMEM without glucose, L-glutamine, phenol red, sodium pyruvate, sodium bicarbonate | Sigma-Aldrich | Cat#D5030 |
| SuperSignal West Pico PLUS Chemiluminescent Substrate | Thermo Scientific | Cat#34580 |
| iTaq Universal SYBR Green Supermix | Bio-Rad | Cat#1725124 |
| TRIzol Reagent | Ambion | Cat#1559018 |
| Phenol:Chloroform:Isoamylalcohol, 25:24:1 | Ambion | Cat#AM9732 |
| RIPA Buffer | Thermo Scientific | Cat#89900 |
| cOmplete Mini Protease Inhibitor Cocktail | Sigma-Aldrich | Cat#11836153001 |
| Vancomycin Hydrochloride from Streptomyces Orientalis | Sigma-Aldrich | Cat#V2002 |
| Neomycin, Sulfate | Fisher Scientific | Cat#BP2669-25 |
| Cefoperazone Sodium Salt | Sigma-Aldrich | Cat#C4292 |
| Ketathesia, 100 mg/mL | Henry Schein | Cat#11695-0702-1 |
| Xylazine Injection, 20 mg/mL | Akorn Animal Health | Cat#59399-110-20 |

| | | |
|---|---|---|
| 2-Metcaptoethanol BP176-100 Electrophoresis | Fisher Scientific | CAS#60-24-2 |
| Bovine Serum Albumin | Sigma-Aldrich | Cat#A7906 |
| **Critical Commercial Assays** | | |
| Quant-iT PicoGreen dsDNA Assay kit | Invitrogen | REF#P7589 |
| Glycogen Assay Kit II (Colorimetric) | Abcam | Cat#ab169558 |
| Ultra-sensitive Insulin ELISA | ALPCO | Cat#80-INSMSU-E01 |
| Bicinchoninic Acid (BCA) Protein Assay | Thermo Scientific | Cat#PI23228 |
| Autokit Glucose | FUJIFILM Medical Systems USA | Cat#99703001 |
| **Deposited Data** | | |
| 16S rRNA amplicon raw sequencing reads | NCBI BioProject | https://www.ncbi.nlm.nih.gov/bioproject/; Accession#PRJNA815335 |
| mRNA raw sequencing reads | NCBI GEO | https://www.ncbi.nlm.nih.gov/geo; Accession#GSE184303 |
| **Experimental Models: Cell Lines** | | |
| n/a | | |
| **Experimental Models: Organisms/Strains** | | |
| Mouse: *Bmal1*$^{flox/flox}$ (B6.129S4(Cg)-*Arntl*$^{tm1Weit}$/J) | The Jackson Laboratory | Strain#007668 RRID#IMSR_JAX:007668 |
| Mouse: Albumin-Cre (B6.Cg-*Speer6-ps1*$^{Tg(Alb-cre)21Mgn}$/J | The Jackson Laboratory | Strain#003574 RRID#IMSR_JAX:003574 |
| Mouse: Cami-Cre (B6 background) | Dr. Joseph Takahashi | n/a |
| **Oligonucleotides** | | |
| 16S_F: 5'-GTGYCAGCMGCCGCGGTA-3' | Earth Microbiome Project | 210570681 |
| 16S_R: 5'-GGACTACNVGGGTWTCTAAT-3' | Earth Microbiome Project | 210570682 |
| **Recombinant DNA** | | |
| n/a | | |
| **Software and Algorithms** | | |
| Graphpad Prism 9 | Graphpad software | https://graphpad.com/scientific-software/prism |
| Empirical JTK_CYCLE | Hutchison *et al.* 2015 | https://github.com/alanlhutchison/empirical-JTK_CYCLE-with-asymmetry |
| QIIME2 | Hall and Beiko, 2018 | https://qiime2.org |
| ImageJ | Schindelin *et al.* 2015 | imagej.net |
| Adobe illustrator CC2020 | Adobe | https://www.adobe.com/product/photoshop.html |
| Orange3 | Demsar *et al.*, 2013 | https://orangedatamining.com |
| CircWave V1.4 | Roelof A. Hut *et al.* | https://euclock.org/results/item/circ-wave.html |

| | | |
|---|---|---|
| Metascape | Zhou *et al.,* 2019 | http://metascape.org |
| Cytoscape v3.8.1 | Cline *et al.*, 2007 | https://cytoscape.org/ |
| STAR v2.6.1b | Dobin *et al.*, 2013 | https://github.com/alexdobin/STAR |
| DESeq2 | Love *et al.*, 2014 | https://github.com/mikelove/DESeq2 |
| fGSEA | Sergushichev *et al.,* 2016 | https://github.com/ctlab/fgsea |
| **Other** | | |
| JL Rat and Mouse/Auto 6F | LabDiet | Cat#5k67 |
| 0.1-mm-diameter zirconia/silica beads | BioSpec Products | Cat#11079101Z |
| Accu-Check Compact Plus Diabetes Monitoring Kit | Roche Diabetes Care | Cat#RO0510-01 |
| OneTouch Blood Glucose Monitoring System | OneTouch | Cat#024046 |
| Mini-PROTEAN TGX Stain-Free Gel | Bio-Rad | CAT#: 4568093 |
| Immobilon PVDF Transfer Membrane | Millipore | IPFL00010 |
| Heparin-coated microvette tubes | Sarstedt | Cat#16443100 |
| 6-well collagen-coated plates | Thermo Scientific | Cat#LS004188 |
| Accucheck Compack Test Strips | Roche Diabetes Care | Cat#FBA_TM82070 |
| OneTouch Ultra Blood Glucose Test Strips | OneTouch | Cat#LI020-994 |
